# SERS‐Based Nano‐ and Microsystems Toward Biomedical Applications

**DOI:** 10.1002/smll.73304

**Published:** 2026-04-15

**Authors:** Gohar Soufi, Tijana Maric, Isidro Badillo‐Ramírez, Juliane Fjelrad Christfort, Matteo Tollemeto, Carmen Milan Guimera, Casper Nisula, Giulia Zappalá, Laura De Vittorio, Marta Rubio‐Huertas, Sungyeong Kim, Ziqiao Li, Morten Bo Søndergaard Svendsen, Zhongyang Zhang, Anja Boisen

**Affiliations:** ^1^ Center For Intelligent Drug Delivery and Sensing Using Microcontainers and Nanomechanics (IDUN) Department of Health Technology Technical University of Denmark, Lyngby Kongens Denmark

## Abstract

Surface‐enhanced Raman spectroscopy (SERS) has emerged as a transformative analytical technique for biomedical and bioanalytical sensing, offering molecular specificity and single‐molecule sensitivity in biological environments. Recent advances in plasmonic nanomaterials, precision fabrication, and compact optical instrumentation have accelerated the development of SERS platforms with expanding potential toward biomedical applications. Despite extensive studies, clinical translation remains limited, underscoring the need for a systematic evaluation of SERS nanomaterials and device architectures under biomedically relevant conditions. This review provides a current overview of nano‐ and microscale SERS‐based devices reported since 2020, encompassing all aspects from material design to practical applications. We highlight controlled SERS‐active architectures, including patterned substrates, nanorods, microspheres, micromotors, and microneedles, as well as combinations of these microfluidic systems. We summarize key studies showing fabrication strategies, instrumentation, and analytical performance with an emphasis on platform capabilities. We present the first focused discussion on static and dynamic SERS platforms for direct, on‐site molecular detection in model biological environments, and outline key challenges for biomedical translation, including reproducibility, sample handling, standardization, and integration. This review provides a practical guide for designing SERS‐based nano‐ and microsystems toward biomedical sensing applications, while clarifying current limitations and future opportunities for translation.

## Graphical abstract

This review provides a current overview of nano‐ and microscale SERS‐based devices, encompassing all aspects from material design to practical applications. We highlight controlled SERS‐active architectures, including patterned substrates, nanorods, microspheres, micromotors, and microneedles, as well as combinations of these microfluidic systems. We summarize key studies showing fabrication strategies, instrumentation, and analytical performance with an emphasis on platform capabilities.

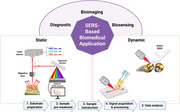

## 1 Introduction

Surface‐enhanced Raman scattering (SERS) spectroscopy is a powerful analytical technique with substantial advantages for sensing, and it is recognized for its broad applicability in bioanalytical and biomedical fields [1, 2]. SERS has found extensive applications in biomedical and bioanalytical sensing, environmental monitoring, and food safety analysis due to its exceptional sensitivity and molecular specificity [3, 4, 5]. SERS enables precise chemical and structural characterization of analytes in biological environments with markedly enhanced sensitivity, reaching the single‐molecule detection level. The integration of advanced nanostructured materials and tailored systems with SERS, alongside innovations in optical instrumentation, has driven significant progress in sensing technologies, facilitating the development of portable and on‐site sensing platforms [3, 6, 7]. Despite numerous examples of demonstrations of proof‐of‐concept SERS‐based sensors in the literature, their advanced implementation in clinical practice remains limited and necessitates further translational research and validation. Many SERS‐active microdevices are first evaluated using well‐defined model analytes to establish sensitivity, signal enhancement, and overall system performance before being extended to biologically relevant applications.

An essential factor for enhancing the performance of SERS‐based sensors is the nanostructured architecture and design employed in the SERS substrate. Recent advances in material nanotechnology, controlled synthesis techniques, and large‐scale fabrication in clean‐room facilities have led to the development of highly robust and reproducible SERS‐active substrates [8, 9]. These substrates may feature diverse morphologies at the nanoscale range, including geometrical patterns, nanorods, microspheres, microparticles, or micro‐nanomotors (MNMs), or designs at the micro/macro scale, such as microneedles (MNs), thereby expanding the application of larger sensing platforms [10, 11, 12]. In such architectures, controlled SERS substrates can be incorporated in a highly controlled manner, enhancing the sensing surface area. The precise design and engineering of these substrates, combined with the intrinsic enhancement capabilities of SERS, enable reliable and sensitive detection of analytes within model and complex biological media. Notably, tailored nano and microscale SERS substrate systems, like microspheres or MNMs, constructed with plasmonic materials demonstrate exceptional sensitivity and spatial resolution for the detection, screening, and imaging of biomarkers and bioanalytes in biological media and systems, even the detection of live cells and bacteria [13, 14, 15, 16].

Recent studies have comprehensively reviewed SERS‐based biomedical applications [17, 18] as well as SERS‐active nano‐ and microstructured architectures, including shape‐tailored colloidal nanostructures, microarrays, and MNMs [6, 11, 19, 20]. However, most of these works have addressed either the material fabrication or the application aspects separately. Nevertheless, it should be emphasized that to evaluate and optimize SERS materials and their sensing performance comprehensively, it is essential to integrate case studies that jointly address both fabrication and application. In contrast to recent reviews which usually have a focus on specific nanostructures or device platforms, this review is structured around the fundamental detection workflow of SERS in biomedical contexts. By distinguishing between static and dynamic substrate paradigms and analyzing their roles across analyte capture, hotspot generation, and signal transduction, we provide a unifying framework that connects material design with functional performance and translational considerations. A critical analysis of relevant factors, including nanosystem design, fabrication methods, instrumental sensing conditions, and analytical performance, is necessary to advance SERS technologies toward realistic biomedical and bioanalytical applications within the current state of the art.

In this review, we aim to present a comprehensive and up‐to‐date overview, including critical discussions and future perspectives, on: (i) The current state of the art in SERS, encompassing its fundamental principles, instrumentation, and recent developments, with particular focus on systems approaching sensor with potential for biomedical applications; (ii) Recent developed SERS micro and nano systems with tailored sensing properties, grouped as static, including nanospeheres or MNs, and dynamic microsystems, including nanomotors. In all these cases, we describe their synthesis and fabrication approaches, along with examples of interesting biosensing applications and proof‐of‐concept bioanalytical applications. This review aims to offer a systematic and up‐to‐date guideline, from material design‐synthesis to application, of novel SERS nano‐ and microsystems for the sensitive detection of bioanalytes with potential biomedical relevance. Moreover, in this work, we present, for the first time, a comprehensive overview of MN arrays with incorporated SERS substrates and their capability for specific and on‐site analyte identification in complex biological media. Furthermore, we provide a critical overview of the challenges, unmet needs, and opportunities to advanced proof‐of‐concept examples of integrated nano‐ and microsystems SERS sensors applications at the point‐of‐care. By targeting researchers in SERS spectroscopy, nanomaterials, analytical chemistry, and biosciences, we anticipate that this review will inspire innovative material designs and expand the scope of biomedical SERS applications.

## 2 SERS and Sensing Principles

This section provides an overview of the fundamental aspects of SERS, including its working principles, classification of substrates, and detailed measurement procedures, to establish the basis for understanding recent advances in microdevice‐ and micromotor‐based SERS systems.

### 2.1 The SERS Principle

SERS is a vibrational spectroscopic technique that significantly amplifies the Raman signal of molecules located in close proximity to a nanostructured metal surface [21]. This enhancement allows for highly sensitive chemical identification based on the unique vibrational fingerprint of each analyte [22].

**FIGURE 1:**
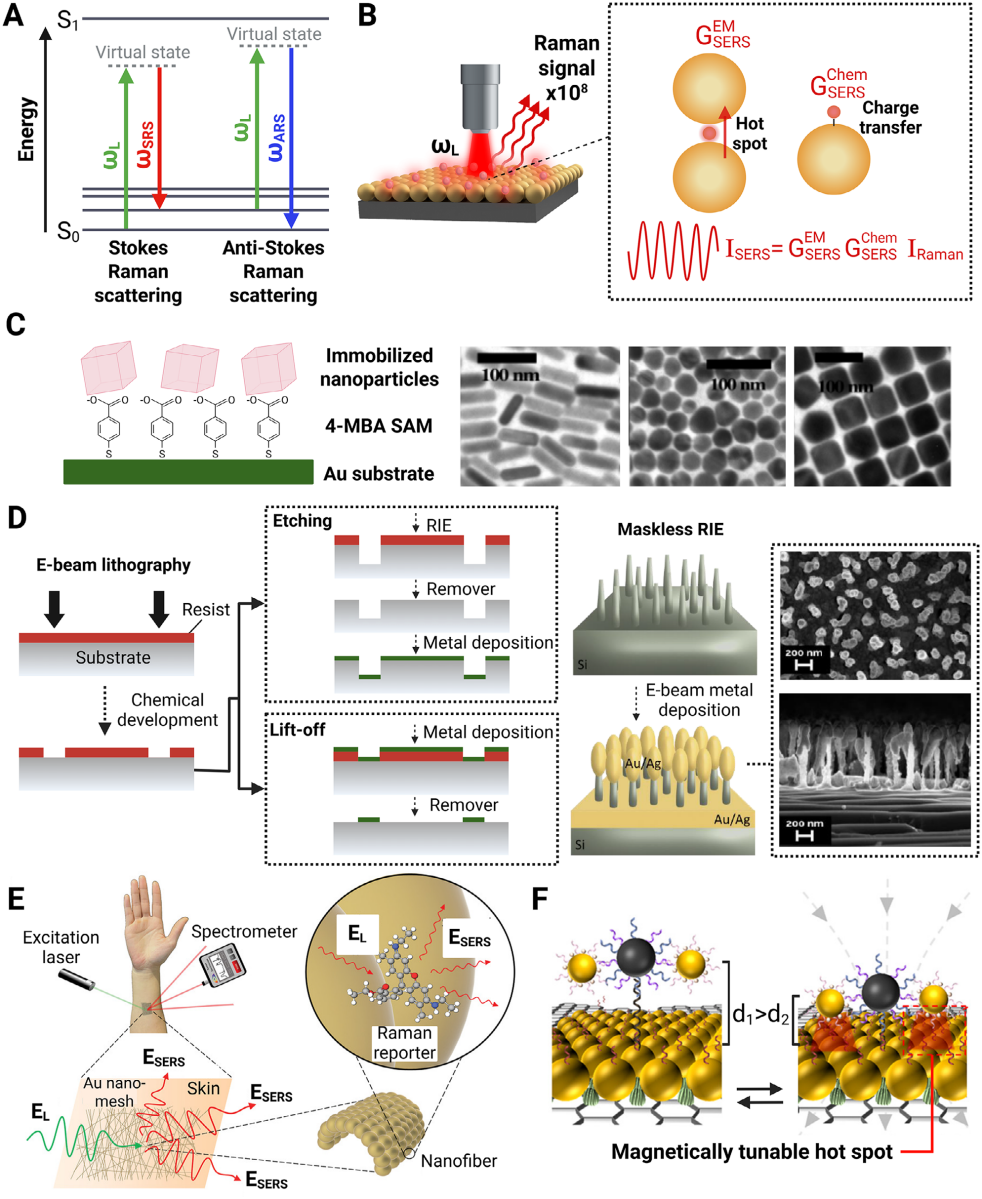
(A) Schematic representation of Surface‐Enhanced Raman Scattering (SERS), illustrating the energy transitions in Stokes and anti‐Stokes Raman scattering, and (B) enhancement mechanisms through electromagnetic (EM) and chemical (Chem) contributions. (C) Functionalization of gold substrates with immobilized nanoparticles and a 4‐mercaptobenzoic acid (4‐MBA) self‐assembled monolayer (SAM) for SERS applications, along with SEM images of nanoparticle arrays. Reproduced with permission [47]. Copyright 2005, American Chemical Society. (D) Nanofabrication approaches using electron beam lithography (E‐beam lithography), reactive ion etching (RIE), and lift‐off techniques for creating structured SERS‐active substrates, with SEM images showing the resulting nanostructures. (E) Wearable SERS on the skin with a nanofiber integrated with gold nanomesh. Reproduced from [57] under the terms of the Creative Commons CC‐BY license. (F) Magnetic‐responsive SERS for selective detection of the N gene of SARS‐CoV‐2.Adapted with permission from [61]. Copyright 2022, American Chemical Society.

The underlying principle of SERS is Raman scattering, a phenomenon first described by C. V. Raman and K. S. Krishnan in 1928 [23]. Raman scattering occurs when a monochromatic radiation source (e.g., a laser) interacts with a sample, causing a photon to interact with a molecule and temporarily distort its electron cloud [24, 25]. This distortion causes an induced dipole moment across a molecular bond, placing it in a short‐lived virtual state due to the oscillating electric field of the light (Figure 1A). As this state decays, the electron returns to the ground state, and Raman scattering results in an inelastically scattered photon with an energy different from that of the incident light [25]. The scattered light intensity can be represented as a spectrum, in which each peak corresponds to a vibrational mode of the analyzed molecule. Since vibrational modes are unique to a molecule's structure, the entire spectrum serves as a distinctive fingerprint for identifying specific compounds [21].

Although Raman spectroscopy is widely used in various analytical fields, its primary limitation is its low sensitivity, as Raman scattering occurs in only about 1 in 10^8^ photons, resulting in a weak signal [26]. To overcome this issue, SERS has been developed to significantly amplify the Raman signal, with enhancement factors (EFs) typically ranging from 10^4^ to 10^8^ and, in some cases, reaching 10^14^ to 10^15^, enabling single‐molecule detection [27, 28].

SERS enhancement arises from two mechanisms: (i) electromagnetic enhancement and (ii) chemical enhancement (Figure 1B) [29].

The electromagnetic enhancement arises from the interaction between incident light and conduction electrons in nanostructured metal surfaces, leading to the excitation of localized surface plasmons (LSPs), observed as localized surface plasmon resonance (LSPR). When the frequency of the incident light matches the resonance condition, strong local electromagnetic fields are generated near the metal surface. In nanoscale regions such as sharp features or interparticle gaps, these fields can become highly concentrated, forming so‐called “hot spots,” which significantly enhance the Raman signal of nearby molecules. Since this enhancement is surface‐confined, it decays exponentially with distance from the metal surface, requiring analyte molecules to be within approximately 1–10 nm to experience significant enhancement [29, 30]. The electromagnetic enhancement is considered the predominant effect responsible for the SERS enhancement, with EFs in the range of 10^4^–10^5^ [31].

The second mechanism contributing to the enhancement is chemical enhancement. It results from the physico‐chemical interaction between the molecule and the metal surface. This involves charge transfer between the metal and the molecule, further enhancing specific Raman modes when the incident light is in resonance with this charge‐transfer transition [32]. Although its contribution is smaller, typically enhancing the Raman signal by a factor of 10^2^ at most, its effect can be combined with the electromagnetic enhancement to further improve SERS sensitivity [33].

Since the discovery of SERS, multiple parameters have been reported to quantify substrate performances. The most rigid definition, known as the SERS substrate enhancement factor (SSEF), was defined by Pablo G. Etchegoin and Eric C. Le Ru in 2007 [34]. This definition expresses EF as the ratio of the Raman signal per molecule under SERS conditions to that under normal Raman conditions, thereby reflecting true molecular enhancement and the intrinsic increase in scattering cross‐section. It is important to note that EFs reported in the SERS literature are calculated using different methodologies, assumptions, and reference standards, which drastically limit direct quantitative comparison across studies [35]. Accurately determining the number of molecules contributing to both SERS and normal Raman signals remains experimentally challenging, often resulting in significant uncertainties. To facilitate practical measurements, the analytical enhancement factor (AEF) was introduced, substituting molecule numbers with analyte concentrations [35]. Although AEF is more accessible experimentally and suitable for analytes initially present in solution or gas phase, it does not fully account for adsorption equilibria and surface transfer effects. This limitation renders AEF sensitive to experimental conditions and can lead to underestimation of intrinsic enhancement. To address these challenges, the SERS performance factor (SPF) was proposed as a sensitivity‐based parameter, defined as the ratio of the slopes of concentration‐dependent SERS and normal Raman calibration curves within their linear regimes [35]. SPF minimizes dependence on specific concentration values and enables a more robust comparison of intrinsic substrate performance, although it necessitates multiple measurements. In summary, EF remains the benchmark for evaluating SERS activity, while AEF and SPF provide practical and sensitivity‐based alternatives, underscoring the ongoing need for rigorous methodology and standardization in SERS performance assessment.

### 2.2 SERS Substrate Classical Classification

The type and chemical composition of the substrate have a significant influence on the performance of SERS [29]. Since its discovery, a wide range of SERS substrates has been developed, with noble metals like gold (Au) and silver (Ag) being the most widely used due to their ability to support LSPR [32]. Other metals, including copper (Cu), aluminum (Al), gallium (Ga), indium (In), platinum (Pt), rhodium (Rh), and metal alloys, have also been explored for SERS applications [32]. More recently, novel materials such as semiconductors [36] and graphene [37], which primarily enhance SERS signals through chemical enhancement rather than plasmonic effects, have been investigated as potential alternatives.

Metallic nanostructures have been fabricated in various shapes, such as nanotubes [38], nanorods [39], and nanocaps [40], using different techniques. Besides sensitivity, several other factors are considered when designing a SERS substrate, including fabrication complexity, cost, reproducibility, scalability, and reusability [41].

SERS substrates are fabricated using various techniques and can be classified as either static (fixed) or active (dynamic) [33]. The former are conventional “stationary” substrates, mounted on a solid support (such as glass or silicon). Common types include colloidal SERS‐active substrates, which are aggregated Au or Ag NPs in solution or drop‐cast onto surfaces (often with the aid of aggregation agents) [33]. These are widely used due to their cost‐effective and straightforward production via the chemical reduction of metal salts [42, 43]. Their size and shape influence plasmonic properties, and while they offer advantages such as heat dissipation and spectral averaging, challenges include uneven distribution, contamination, and spectral variations. The stabilizing agents required to prevent aggregation can also interfere with analyte signals, making careful preparation essential [42, 44]. Another type of static substrates is metal films/nanostructured substrates, including nanostructure arrays such as Nanorods, nanowires, nanopillars, nanohole arrays. These allow more controlled hot spot creation and reproducibility. This approach involves immobilizing metallic nanoparticles onto solid substrates through chemical self‐assembly (Figure 1C), a method known as the bottom–up approach [33, 45, 46, 47]. Bifunctional molecules such as thiols or amines are used to attach nanoparticles, and techniques like layer‐by‐layer (LBL) deposition allow for controlled multilayer formation [48, 49]. Electrostatic self‐assembly [50], chemical growth [51], and vapor deposition [52] are alternative approaches; however, they often encounter issues with production efficiency and uniformity. While these methods improve stability and reproducibility compared to colloidal solutions, they still require careful optimization to achieve high enhancement factors [53]. For greater control over substrate structure and reproducibility, top–down fabrication methods such as nanolithography, nanoimprint lithography, and etching techniques have been employed [33, 54]. Electron beam lithography (EBL) is a widely used nano lithographic method that enables precise control over nanostructure size, shape, and spacing, with resolutions down to the nanometer scale (Figure 1D) [55]. However, while EBL allows for optimization of key plasmonic features, it remains an expensive and time‐consuming technique that requires specialized equipment [33]. To overcome these limitations, alternative methods such as maskless reactive ion etching (RIE) have been developed to fabricate large‐area SERS substrates more efficiently [40]. Laser‐based methods, such as femtosecond and picosecond laser ablation, offer a rapid alternative by creating nanostructured surfaces that are later coated with thin layers of Au or Ag, resulting in stable and uniform SERS substrates with strong EFs [54].

Each fabrication method has its advantages and limitations in terms of cost, reproducibility, and scalability. Research continues to refine these approaches, improving SERS substrate performance for a wide range of applications [41].

On the other hand, there are flexible substrates which include paper, polymer films, textiles, polydimethylsiloxane (PDMS), and films with embedded nanoparticles. These are attractive for low‐cost, portable SERS devices.

Recent reviews discuss progress in micro‐ and nanostructured SERS substrates, including the transition from rigid to flexible, stretchable substrates for wearable and field applications (Figure 1E) [56, 57].

The second class of substrates is active (dynamic) substrates, which are developed to overcome mass transport limitations and improve local analyte delivery [58]. Micromotors/ nanomotors (MNMs) functionalized with plasmonic metals are mobile micro/nanomachines (e.g., catalytic [59], light‐driven [60], acoustic, magnetic) decorated or coated with plasmonic nanostructures. The motion of the motor actively sweeps analytes, delivers them to hot spots, and enables in situ sensing. A growing body of work exists on SERS‐active micromotors (MMs), as further discussed in Section 4. Moreover, there have been development in stimuli‐responsive/reconfigurable substrates (Figure 1F) whose geometry or configuration can be changed via external stimuli (magnetic fields [61], electric fields [62], light actuation [63], mechanical deformation [64] to dynamically control hot spot distribution or reposition analyte capture zones.

### 2.3 SERS Procedure and Analysis

The instrumentation and procedural methodologies employed in SERS are crucial for ensuring accurate measurements and signal enhancement. This section will discuss key components in SERS instrumentation, measurement procedures, and practical considerations for effective application.

**FIGURE 2:**
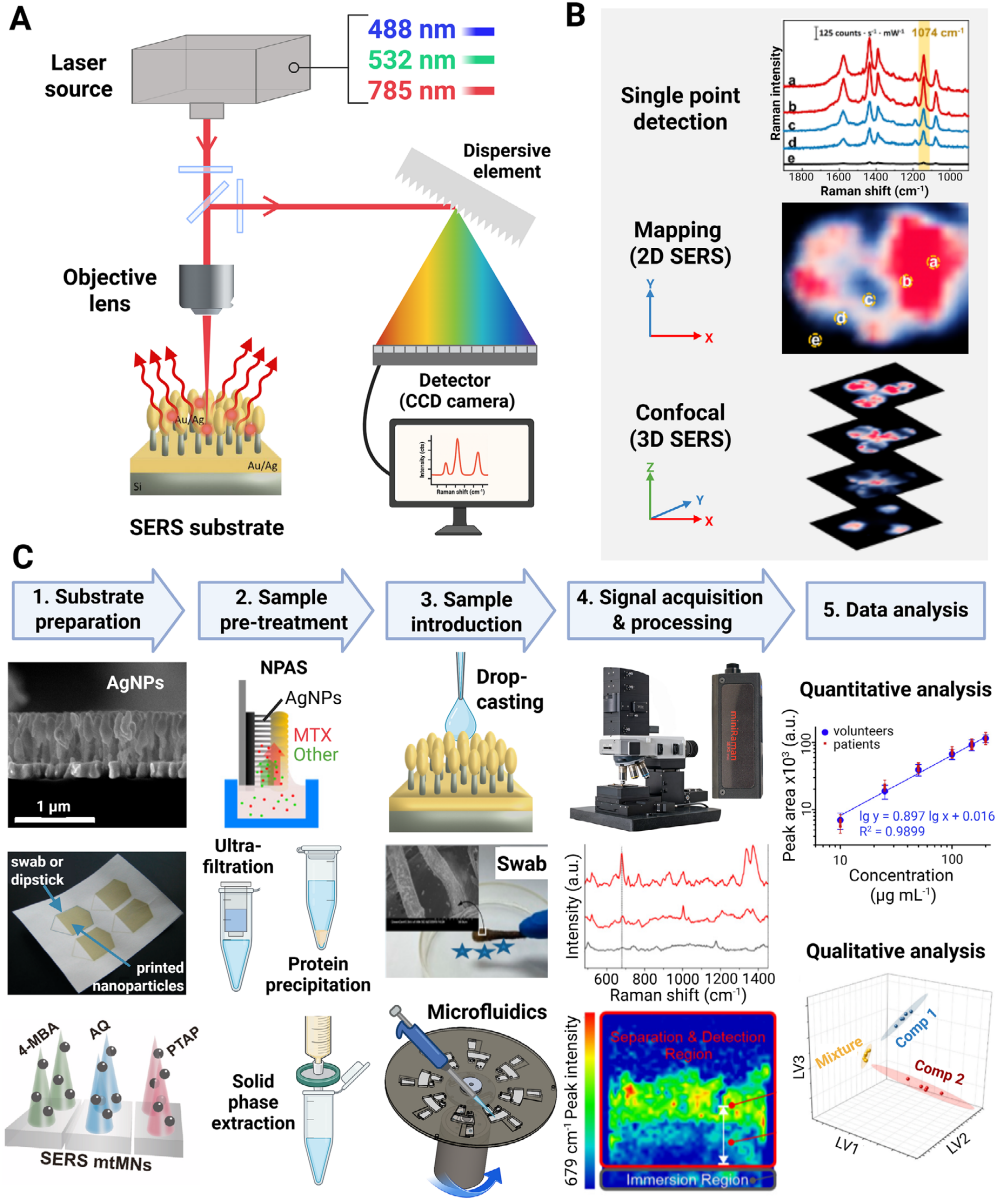
(A) Schematic diagram of SERS instrumentation with beam path and key components. (B) Different types of Raman measurements including single point, 2D mapping, and 3D mapping (confocal). Adapted with permission [46]. Copyright 2022, The Royal Society of Chemistry. (C) Flowchart illustrates the SERS measurement procedure and its main steps: (1) substrate preparation via the suitable method (e.g., reactive ion etching, inkjet printing (reproduced with permission [81]. Copyright 2014, The Royal Society of Chemistry), or nanoparticle‐deposition. (reproduced with permission [15]. Copyright 2023, Elsevier Ltd)); (2) sample pre‐treatment to improve analyte concentration and minimize sample matrix interference, either directly on the substrate (e.g., nanopillar‐assisted separation (NPAS) (reproduced with permission [71]. Copyright 2021, American Chemical Society), or separately (ultra‐filtration, protein precipitation or solid‐phase extraction) (reproduced with permission [72]. Copyright 2024, Elsevier Ltd)); (3) sample introduction by different techniques to achieve analyte adsorption onto the substrate (e.g., drop‐casting, swabbing (reproduced with permission [82]. Copyright 2014, American Chemical Society)), or by microfluidic systems); (4) signal acquisition and processing: the Raman measurements can be conducted using, e.g., table‐top Raman microscopes (e.g. WITec [80]) or hand‐held spectrometers (Lightnovo [83]), followed by spectral and hyperspectral corrections. Reproduced with permission [71]. Copyright 2021, American Chemical Society. (5) data analysis: transition from spectral data to quantitative and/or qualitative results via chemometric methods that allow the identification of components in the sample and their quantification based on previous calibration. Reproduced from [80] under the terms of the Creative Commons CC‐BY 4.0 license; and reproduced with permission [84]. Copyright 2015, Elsevier Ltd.

The primary instrument used in SERS is a Raman spectrometer, which typically consists of a laser source for excitation, an objective lens or probe to focus the laser onto the sample, a spectrometer for dispersing the scattered light, and a detector to record the spectral data (Figure 2A). The choice of laser wavelength is crucial, as it must align with the plasmonic resonance of the employed substrate. Commonly utilized laser wavelengths include 488, 532, or 785 nm, depending on the type of substrate and the specific application.

In SERS detection techniques, the methods of single‐point detection, mapping, and confocal measurements are three main techniques that significantly enhance the capabilities of this analytical tool (Figure 2B). Single‐point (Spot) measurement involves focusing the laser onto a specific location on the SERS substrate and collecting the resulting Raman signal from that point. This technique is particularly advantageous for advanced techniques, such as optical tweezers and plasmonic nanopore systems, which enable the manipulation and detection of individual cells, particles, or even single molecules [65, 66].

Raman mapping (imaging) involves 2D scanning of the sample (*x–y*) and collecting a spectrum at each pixel, thereby assembling a 2D chemical map [65, 66]. This helps to reveal spatial heterogeneity and distributions of analyte/hot spots. A confocal Raman [depth profiling) the setup allows *z*‐axis resolution, reducing background from out‐of‐focus regions. Useful in layered systems or microfluidic channels [46].

Moreover, time‐resolved SERS is a modality that enables a system to conduct measurements at the same location over time, allowing for the monitoring of adsorption/desorption dynamics, chemical reactions, or catalytic processes. Motion‐resolved enabled systems have also been developed, in which the Raman laser may track moving objects in the sample (e.g., MMs) or interact with them as they traverse the focal spot, enabling spatiotemporal sensing [67, 68, 69].

While these advanced measurement techniques offer significant advantages, challenges remain.

SERS measurement procedure typically includes several sequential steps (Figure 2C):

1. Substrate Preparation: The performance of SERS is highly dependent on the properties of the SERS substrates, which must be biocompatible, reproducible, and robust. SERS can be performed using direct detection, where analyte‐specific bands are directly observed, or indirect detection, which relies on the SERS response of a secondary “reporter” molecule stimulated by the analyte's presence. While direct methods provide molecular information without relying on Raman reporters, indirect methods with well‐characterized reporters provide better control, especially in complex biological matrices. The choice of SERS substrate has a significant influence on signal enhancement.
2. Sample preparation: Sample preparation steps have a key role for successful SERS measurement in complex clinical samples like blood, serum, urine, or wounds. In complex matrices, the signal of the desired analyte can be covered by the other present components. Recent research shows that SERS substrates can both boost the signal and serve as a sample treatment. For instance, nanopillar‐assisted separation (NPAS) uses nanostructured SERS surfaces to separate and analyze different components in biological fluids like serum and urine [70, 71]. The NPAS method combines separation and signal enhancement, which helps reduce interference from substances like urea or creatinine. Filter paper‐based SERS substrates and other composite materials can also filter samples while enhancing Raman signals, cutting down on particle interference without the need for extensive off‐line treatment.

In addition to using special substrates, several standard and advanced sample preparation methods have been adapted or combined with SERS to improve sensitivity and selectivity in complex samples. These methods include dilution, protein precipitation (PP), and ultrafiltration (UF) to remove large interfering molecules [71]. Other techniques, like solid‐phase extraction (SPE) [72, 73], liquid–liquid extraction (LLE) [74], and supported liquid membrane (SLM) [75], help concentrate low‐level analytes and remove unwanted substances before analysis. By combining sample preparation and enrichment with SERS detection, either through substrate design or earlier processing steps, researchers can make results more reliable and reduce variation in clinical samples.

3. Sample Introduction: Efficient analyte delivery to the hot spots is critical, especially in micro/nanoscale systems where diffusion is limited. The analyte of interest is introduced to the SERS substrate through various means, such as direct deposition, like droplet casting [76] or immersion [76], introducing via microfluidics or via swab techniques [7, 77], as demonstrated in studies for detecting trace explosives. In the case of MNMs, these may actively capture analytes (via chemical affinity or electrostatic interaction) and transport them to detection zones or sweep the system with SERS‐active surfaces [58].
4. Raman acquisition: The objective lens collects the scattered light post‐excitation, which is then filtered to eliminate Rayleigh scattering before being directed to the spectrometer. As mentioned previously, there are different types of measurement methods. In many applications, including biodetection, the high spatial resolution provided by micro‐spectroscopy techniques is advantageous. Instruments like the Renishaw inVia Raman microscope and WITec are often employed for high‐resolution spatial mapping and quantitative measurements, showing comparable results to other methods while achieving satisfactory reproducibility [7, 76]. Additionally, portable Raman spectrometers have emerged, allowing for field applications and on‐site analysis, which significantly enhances the practical utility of SERS in diverse environments [65]. After excitation, the scattered light is collected through the objective lens, passed through optical filters to eliminate Rayleigh scattering, and then directed into the spectrometer [78].
5. Data analysis: The obtained Raman spectrum is analyzed for specific molecular fingerprints that can be correlated with the presence and concentration of the target analytes. Baseline corrections are often applied to the spectral data to account for background noise, ensuring that the resulting intensity reflects the actual signal from the analytes of interest [78, 79]. SERS is widely recognized as a versatile analytical technique that integrates both qualitative and quantitative approaches in its applications, particularly within biomedical sciences [78, 79]. For qualitative analysis, SERS spectra provide molecular fingerprints, enabling the identification of specific chemical components in complex biological matrices. Beyond identification, it also enables the characterization of protein structures, monitors chemical interactions, and classifies disease states. When it comes to quantitative analysis, SERS aims to determine analyte concentrations, a task that has seen significant advancements despite challenges like signal fluctuations. Strategies for quantification include calibration curves, the use of various internal standards (e.g., principal component analysis (PCA), hierarchical cluster analysis (HCA) for unsupervised learning; partial least squares (PLS), support vector machines (SVM), neural networks (NN) for supervised learning [7, 76, 77, 80], are critical for extracting precise qualitative and quantitative information from complex SERS data. This dual capability makes SERS a powerful and versatile tool in various fields, from fundamental research to practical diagnostic applications.

Moreover, the combination of SERS with MNMs, microcapsules, and microdevices marks a paradigm shift in analytical chemistry, where the integration of advanced detection techniques with innovative microengineering opens pathways for the development of highly sensitive biosensing applications. These technologies represent crucial steps toward improving the detection and analysis of low‐concentration analytes in various environments, ultimately enhancing the effectiveness of diagnostics and biosensing applications.

After discussing all the main steps involved in SERS measurements, from substrate preparation and sample introduction to signal detection and data analysis, we now turn our focus to the substrates, which play a central role in determining sensitivity and reproducibility. In the following sections, we classify SERS substrates into two main categories: static systems, in which the SERS‐active structures remain fixed during analysis, and dynamic systems, in which the SERS‐active entities themselves undergo motion or respond to external stimuli or self‐propel, enabling active analyte transport and mixing.

## 3 Static SERS‐Active Microsystems for Bio‐Sensing Applications

Static SERS platforms enhance signal through predefined structural architectures, rather than active motion, to concentrate fields and regulate analyte transport. We first examine microspheres/microcapsules as versatile carriers and scaffolds that stabilize hot spots and enhance reproducibility, then proceed to microneedles, which couple these plasmonic architectures with painless dermal access.

### 3.1 Microspheres/Microcapsules

Microspheres provide a highly effective platform for SERS due to their ability to enhance Raman signal uniformity, improving reproducibility, and facilitating controlled analyte interactions. The unique structural advantages of microspheres, including their high surface area, uniform analyte distribution, and ability to integrate diverse materials, make them an ideal choice for SERS‐based sensing applications. Spherical shapes are relatively simple to produce compared to other complex geometries, as their formation is often driven by natural thermodynamic processes such as surface tension minimization in emulsions or self‐assembly mechanisms. Their shape helps enhance Raman signals by promoting uniform electromagnetic field enhancement and the formation of plasmonic hot spots on the curved surface, while also allowing the incorporation of different functional materials. Several fabrication approaches exist for creating SERS‐active microspheres. One method, as mentioned earlier, is nanoparticle aggregation, where colloidal nanoparticles cluster into microscale assemblies, creating strong plasmonic coupling that enhances the SERS effect; however, achieving uniform aggregation can be challenging. Another approach is template‐assisted synthesis, where microspheres are formed using templates such as porous silica or polymer beads, allowing for precise control over size, porosity, and surface properties. Porous templates offer high surface areas for analyte adsorption and can encapsulate functional molecules for applications such as drug sensing. This technique enables the integration of otherwise difficult‐to‐incorporate materials, such as lipids. Last, template removal involves using sacrificial templates, such as polystyrene (PS) beads or emulsions, which are later dissolved or calcined to create hollow or porous microspheres. These microspheres can incorporate materials like responsive polymers, thereby enhancing analyte specificity.

The incorporation of microspheres into SERS platforms has further enhanced their effectiveness by improving sensitivity, reproducibility, and stability in complex biological and environmental matrices. These microspheres, typically composed of hydrogel matrices, polymers, or multilayered shells, serve as structured environments that regulate analyte interaction with embedded SERS‐active nanomaterials, optimizing detection efficiency and reliability [1, 85]. Microspheres are found to be efficient SERS substrates for in situ detection of small toxic molecules in complex real samples, such as biological fluids and food. Representative examples of SERS‐active microcapsules and microspheres, along with their corresponding biomedical applications, are summarized in Table 1.

**TABLE 1:** Representative microcapsule‐ and microsphere‐based SERS systems toward biomedical applications.

Material	Size and physicochemical properties	Sample matrix	Target molecule	Analytical performance	Refs.
Toluidine blue‐loading DNA microcapsule	Uniform porous spheres size 1–2 µm	Aqueous suspension (calibration) 1000× diluted human serum (validation)	miRNA	Detection range: 1 fm–10 nm LOD: 6.7 × 10^−16^ m recoveries: 93.9%–101.4% RSD: 0.4%–2.2%	[14]
(Semipermeable alginate microparticles encapsulating AuNPs	Microparticles 100‐200 µm semipermeable shell AuNPs incorporated	Aqueous suspension (calibration) human serum without protein removal (validation)	6‐TG	Detection at 1 × 10^−6^ m 6‐TG in the presence of 1 × 10^−3^ m BSA; selective SERS response due to size/charge permeability	[86]
(Microgels containing highly concentrated AuNPs	Microgels ∼89 µm diameter AuNPs 20 nm	Distilled water (calibration) Milk (validation)	Tricyclazole	LOD: 1 ppb in milk (below regulatory threshold) and 1 ppb in distilled water	[87]
(SERS‐hydrogel micropellet	Micropellets ∼49 µm pore size: 5–12 nm AuNPs@4‐MBN: 7 nm AgNPs ∼40 nm (after silver mirror reaction)	Aqueous solution (calibration) whole blood (validation for glucose) milk (validation for melamine)	Glucose and melamine	For glucose: detection range: 0.1–20 mm in whole blood LOD: 0.01 mm RSD: 3.41%. for melamine: detection range: 10 nm–1 mm in milk LOD: 10 nm RSD: 7.67%	[88]
(Porous Au microparticles (dual ex situ AuNP adsorption and in situ HAuCl_4_ reduction on CaCO_3_ templates)	Spherical microcrystals ∼11 µm pore size: 5–30 nm AuNPs 19 ± 9 nm	Aqueous suspension (calibration)	Rhodamine B (probe molecule) BSA (model protein in complex media)	EF: 7.6 ± 1.6 × 10^8^ for BSA EF: 2 × 10^8^ for Rhodamine B enhancement 3‐5 orders of magnitude higher than comparable scaffolds	[89]
(Fe_3_O_4_@SiO_2_ microparticles with Au nanospikes	Composite microparticles ∼380 nm magnetic core–shell structure with dense Au nanospikes	Lake water, juice, and soil (validation)	Thiram	LOD: 1 nm for thiram directly in complex samples rapid SERS detection without pretreatment	[90]
(Au and Ag all‐nanoparticle microcapsules	Microcapsules 4.25 ± 0.82 µm Au/Ag multilayer (6–8 layers)	Aqueous suspension (calibration)	E. coli	EF: up to 7.8 × 10^4^ (1.5 mW laser) and 2.8 × 10^3^ (5 mW laser) Enabled detection of 13 additional bacterial peaks beyond standard fingerprint signals	[91]
(AuNPs agglomerates and aggregates	Not reported (NP clusters formed by agglomeration/aggregation)	Aqueous suspension (calibration)	CPF	Detection range: 0.001–1 ppm LOD: 0.009 ppm RSD: 11.83%	[92]

Abbreviations: RSD: Relative standard deviation. LOD: Limit of detection 6‐TG: 6‐thioguanine BSA: Bovine serum albumin CPF: Chlorpyrifos *E. Coli*: *Escherichia coli*

**FIGURE 3:**
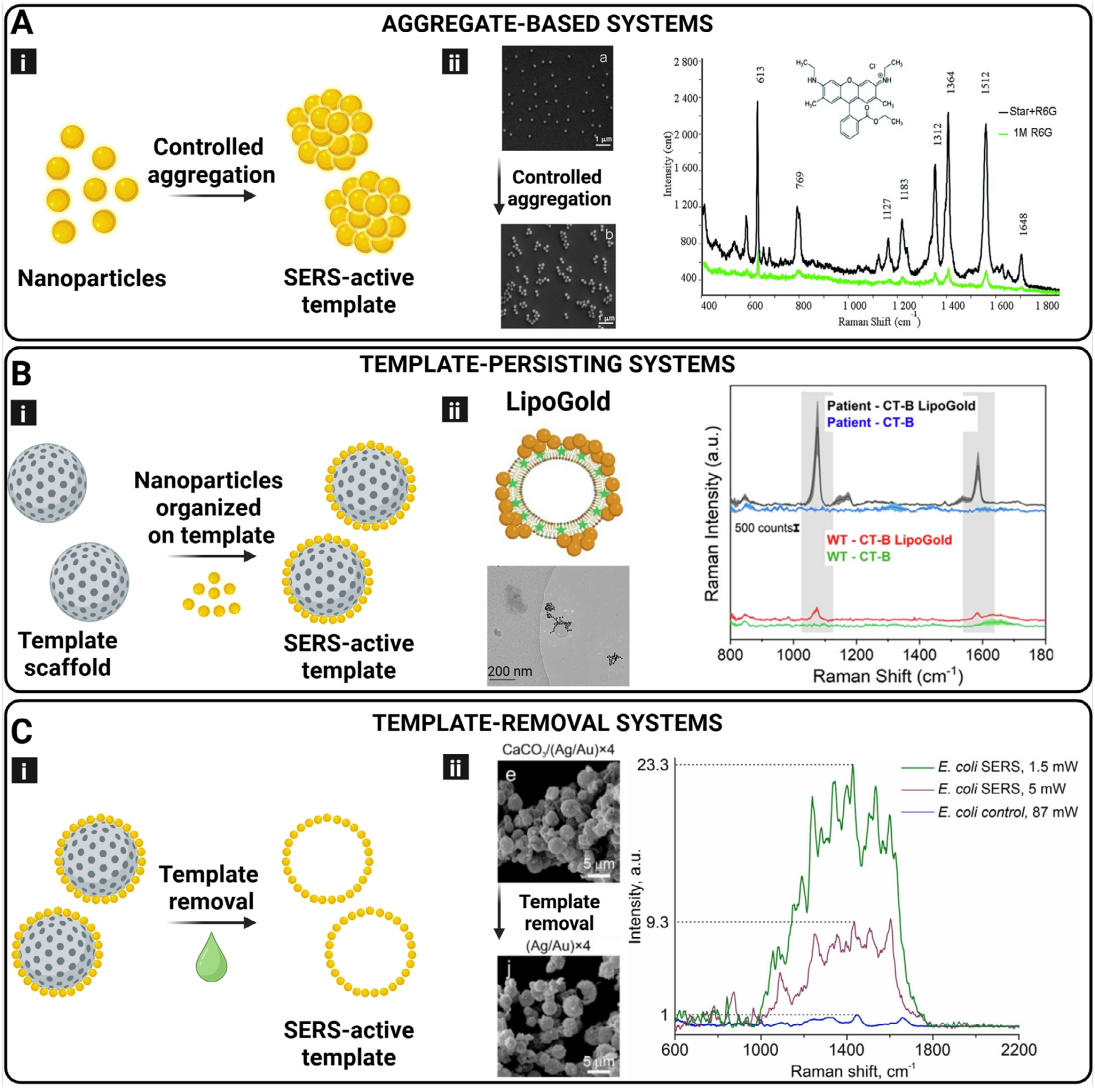
Fabrication and application of microsphere‐based SERS systems. (A) (i) Schematic illustration of aggregate‐based systems. (ii) Application example showing SEM images of microspheres before and after aggregation, together with SERS intensity at different laser powers and detection of rhodamine. Reproduced with permission from [43]. Copyright 2014, The Royal Society of Chemistry. (B) (i) Schematic illustration of template‐persisting systems. (ii) Application example showing the fabrication of lipid‐based gold nanoparticle–coated substrates (Lipogold) with corresponding cryo‐TEM images. SERS spectra are shown for the wild type in the presence or absence of Lipogold, as well as Raman–SERS spectra for patient samples affected by juvenile GM1 gangliosidosis. A histogram illustrates the quantitative Raman intensity of GM1 at 1060 cm^−^^1^. Reproduced from [109] under the terms of the Creative Commons CC‐BY 4.0 license. Copyright 2024, Springer Nature. (C) (i) Schematic illustration of template‐removal systems. (ii) Application example using calcium carbonate templates coated with silver and gold nanoparticles, with corresponding SEM images. SERS detection of rhodamine and E. coli is also presented. Reproduced with permission from [91]. Copyright 2024, Springer Nature.

As mentioned above, several methods and materials are employed for the fabrication of SERS‐active microspheres. For brevity, we will explicitly divide the various systems into three groups: aggregate‐based systems, template‐persisting systems, and template‐removal systems. An illustration of the different systems is shown in Figure 3.

#### 3.1.1 Aggregate‐Based Systems

SERS conventionally employs small, colloidally stable noble metal nanoparticles, most commonly gold (Au NPs) or silver (Ag NPs), to amplify Raman signals from nearby analytes [43, 93]. A common route for synthesizing these nanoparticles involves the chemical reduction of metal salts, typically chloroauric acid (HAuCl_4_) for gold or silver nitrate (AgNO_3_) for silver, in aqueous solution using reducing agents such as sodium citrate, ascorbic acid, or sodium borohydride [94, 95]. Stabilizing or capping agents (e.g., citrate, CTAB, or polyvinylpyrrolidone (PVP)) are often introduced to prevent aggregation and to help control particle morphology, size distribution, and colloidal stability. By tuning parameters such as temperature, pH, reagent concentrations, and reaction time, researchers can reproducibly generate nanoparticles with defined shapes (spheres, rods, cubes, stars) and optical properties tailored for SERS applications [94, 95]. These particles, typically in the 20–60 nm size range, are favored for their well‐defined LSPR properties and ease of functionalization. [10] For anisotropic Au NPs, two primary LSPR modes are typically observed: the transverse and longitudinal plasmon bands. While the transverse mode is largely influenced by particle size, the longitudinal mode is highly dependent on the aspect ratio, defined as the ratio of the longest to the shortest dimension of the particle [96, 97]. One such example from Feng et al. synthesized Au NPs, which composed the nanoplates, via a one‐pot seedless method [10]. The nanoparticles were fabricated with different surface morphologies by modifying the amount of 2‐mercaptobenzoimidazole‐5‐car­boxylic acid (MBIA), the structure‐directing reagent. An increase in the MBIA concentration leads to a rougher surface of the Au NPs as well as to an increase in the SERS intensity. The largest enhancement was demonstrated on Au NPs, characterized by large‐sized vertically aligned thin nanoplates. Even though the use of MBIA allows for high SERS sensitivity, the strong interaction with the ligand prevents molecules with weak surface affinity, such as Rhodamine 6G (R6G) and Methylene Blue (MB), from attaching to the surface of the Au‐NPs. However, over time, it has become increasingly evident that single nanoparticles provide limited enhancement compared to systems where plasmonic “hot spots” are generated through controlled aggregation or clustering. This realization has led to the development of aggregated NP systems, engineered assemblies where interparticle gaps dramatically boost local electromagnetic fields, thereby significantly increasing SERS sensitivity (Figure 3A).

Aggregation‐based systems are defined by their ability to form the clusters required for SERS without any covalent bonding between or within the single units constituting the SERS substrate. Aggregation is typically induced through simple physicochemical triggers that promote interparticle interactions and hot spot formation. Common strategies include the addition of electrolytes such as sodium chloride (NaCl), potassium chloride (KCl), or magnesium sulfate (MgSO_4_) to screen surface charges and reduce electrostatic repulsion, as well as pH changes, solvent exchange, or polymer‐induced bridging using agents like polyethylene glycol (PEG) or polyvinylpyrrolidone (PVP) [94, 95]. These methods facilitate the controlled formation of nanoparticle clusters while preserving their colloidal nature. Tian et al., investigated the ability of Au NPs of various geometrical shapes to act as SERS substrates for biomedical applications, with the aim of establishing optimization parameters for SERS design toward biomedical sensing by comparing critical conditions and material properties. Three different shapes were produced: nanospheres, nanotriangles, and nanostars. The SERS samples were investigated over a concentration range of 0.1–10 µm of R6G and compared to the spectra of R6G alone in a 1 m aqueous solution to enable systematic comparison of aggregation and morphology effects. The study demonstrated that the SERS signal is enhanced by both the aggregation of nanoparticles and the roughness of the nanoparticle surface. Aggregation of AuNPs was induced by pH modification of the solution. The surface plasmon resonance (SPR) excitation for isolated spheres could be determined as negligible, whereas the curved nanoparticles provided a strong excitation regardless of aggregation. The increasing effect was ordered: nanospheres < aggregated nanospheres < nanotriangles < nanostars [43]. Similarly, Liu et al., prepared spherical AuNP colloidal solutions consisting of approximately spherical particles with the purpose of using SERS to detect chlorpyrifos (CPF, an agricultural pesticide toxic to humans, in cucumbers. CPF itself was shown to induce agglomeration and aggregation of AuNPs. This leads to an interesting SERS signal relationship, where the SERS signal follows a linear relationship from 0.001 to 1 ppm of CPF, with a LOD of 0.009 ppm, but exhibits the inverse at concentrations higher than 1 ppm of CPF. By combining analysis of calorimetric response, absorbance spectra, and PCA, the presence of CPF could be detected in real samples with an LOD of 0.11 ppm and an RSD of 11.83% [92]. These studies demonstrate how aggregates and agglomerates of AuNPs can be tuned through sample solvent modification, by the amounts of the reagents, or by the intrinsic concentration of the analyte that is being detected through SERS.

#### 3.1.2 Template‐Persisting Systems

This method refers to SERS platforms in which plasmonic nanoparticles are organized or synthesized directly onto a physical scaffold that remains part of the final structure (Figure 3B). These templates provide structural integrity and spatial control over nanoparticle distribution, which is critical for generating and maintaining consistent plasmonic hot spots [98]. Templates can be fabricated through one‐pot synthesis approaches, employing materials such as silica particles or calcium carbonate particles, where the template and nanoparticle assembly occur simultaneously in solution [99, 100, 101]. In the case of silica, this typically involves sol–gel chemistry, most commonly via the Stöber process, where tetraethyl orthosilicate (TEOS) undergoes hydrolysis and condensation to form spherical silica templates [13]. For calcium carbonate particles, on the other hand, the formation occurs through simple inorganic precipitation reactions (e.g., by mixing calcium and carbonate salts) [102]. Both systems provide versatile platforms for templated nanoparticle fabrication depending on the desired material properties and process conditions.

Alternatively, microfluidic‐based methods offer greater precision in controlling both the geometry of the template and the spatial arrangement of nanoparticles, by directing the flow and self‐assembly of materials within microscale channels [87, 103]. Microfluidic chips integrate components like microchannels, micromixers, micropumps, and valves to perform sample mixing, reaction, separation, and detection in one compact system, offering advantages such as low sample and analyte consumption, fast reactions, and high reproducibility [104, 105]. These methods often employ softer, more tunable materials such as hydrogels, emulsions, or polymer matrices, which contrast with the more rigid and inorganic scaffolds typically used in one‐pot syntheses (e.g., porous silica or calcium carbonate). Because the template is not removed after synthesis, it also serves as a structural backbone, enabling consistent performance across different batches and making the system more robust for practical sensing applications. For example, in a study by Mukherjee et al., one‐pot synthesis was used to produce silica microspheres (SMPs) functionalized with mercapto‐groups and covered with AgNPs and AuNPs. The mercapto‐groups act as immobilizers for the NPs. AgNP deposition was done by Tollens’ reagent, whereas AuNP deposition was accomplished through sputtering. Notably, the homogeneity of AuNP distribution on the surface of SMPs was higher than the homogeneity of AgNPs. The analyte used for SERS analysis was MB. For AgNPs and AuNPs, an SERS EF, based on the ratio $EF=\frac{I_{hotspot}}{I_{substrate}}$, with a 633 nm excitation laser, was calculated to reach up to 24 and 26, respectively [106, 107, 108]. Cardellini et al., demonstrated a method for clustering AuNPs via self‐assembly on lipid vesicles (LipoGold). By including Raman reporters (RRs) in the lipid membrane via hydrophobic interactions and controlled clustering of AuNPs on the surface of the liposome, the RRs could freely diffuse to the clusters and form responsive hot spots for SERS. Functionalization with antibodies allowed for the detection of intracellular GM1 alterations using SERS. The liposomal membrane of the vesicle could be modified with the RRs4‐Mercaptobenzoic acid (4‐MBA), 4‐nitro‐N‐(2‐mercaptoethyl)benzamide (RR_2_), or 4‐(phenylethylnyl)‐N‐(2‐mercaptoethyl)benzamide (RR_6_). Additionally, by pegylating the membrane, antibodies of various types could be conjugated to the membrane through EDC/NHS coupling after self‐assembly of AuNPs on the membrane, leading to tunable targeting of the liposomes. To determine GM1 alterations, skin biopsies of a healthy subject and a GM1 gangliosidosis patient were analyzed. Fibroblasts from both were incubated with equivalent amounts of cholera toxin subunit b‐fitc(CT‐B) or CT‐B‐LipoGold and fluorescent intensity was measured through flow cytometry. The difference in Raman signal between healthy and diseased cells, shown by the LipoGold probes, shows promising results for use as a tool in detecting GM1 alterations [109].

To develop SERS substrates for biomedical applications, Hinds et al., produced AuNP composites through two different microsphere‐assisted template‐persisting methods [110]. The difference between the methods is the assembly onto the porous carbon particle. In one method, the carbon microspheres were oxidized with nitric acid to attach a layer of ─COOH groups. This allowed for electrostatic AuNP‐dimethylaminopyridine (DMAP) assisted seeding of carbon microspheres. Finally, by treating the seeded particles with HAuCl_4_ and hydroquinone (HQN), the particles were ripened to a gold “shell”. In the second method, the oxidized particles were converted to amide‐linked cystamine through EDC/NHS coupling. In contrast to the previous method, the particles were seeded with borohydride‐capped AuNPs (AuNP (BH)), which gave a sparser layer of AuNPs. Finally, the particles were also treated with HAuCl_4_ and HQN. Both methods resulted in a porous carbon particle being converted into an AuNP carbon composite. Comparatively, the shell of the AuNP (BH) treated carbon microparticles (Au: C ratio 7.0) was less densely populated with AuNPs than the AuNP‐DMAP treated particles (Au: C ratio 1.6). The SERS activity was determined by optical trapping Raman microscopy due to background signal reduction. Malachite green (MG) was used as the model probe due to its documented Raman profile as well as its presence in fish stock as a contaminant. Despite the work focusing on detecting MG, the authors suggest that the particles are expected to be suitable for a variety of analytes that can be chemisorbed or physisorbed at the surface, thereby providing possibilities for biomedical applications.

In another study, magnetic microparticles covered with Au nanospikes demonstrated fast detection of the pesticide thiram in lake water, juice, and soil, with strong SERS signals down to 1 nm [90]. After dispersion of the microparticles in real samples, the pesticide is captured through strong Au─S bonds without any pretreatment in juice and water, or by extraction with ethanol in the soil sample. Finally, the SERS substrates are easily separated from the solution using a magnet. These results show promise toward rapid and low‐cost on‐site preliminary analysis of real samples.

##### 3.1.2.1 Microfluidic Synthesis

Synthetic approaches through microfluidics provide a powerful strategy for the production of hydrogel monodisperse microparticles, with uniform size, shape, and surface properties [104, 105]. These microspheres can be concentrated with metal nanoparticles to achieve high SERS sensitivity. Kim et al. presented microgels loaded with Au NPs produced with a glass capillary microfluidic device. In this study, Au NPs are concentrated in a water‐in‐oil‐in‐water (W/O/W) double emulsion at hypertonic conditions, without aggregation and with an average interparticle distance as small as 48 nm. This approach enables the detection of tricyclazole, a fungicide used to control rice blast, directly in whole milk [87]. Here, the matrix of the microgels rejects the large water‐soluble proteins contained in milk while allowing the permeation of small molecules, like tricyclazole, without sample pretreatment. The system shows a high and uniform Raman intensity because of the even distribution of nanoparticles within the microgels, with a LOD estimated as 1 ppb, lower than the allowable level of the pesticide in milk.

To develop a fast, low‐cost, and reliable method for measuring glucose in whole blood without sample pretreatment, Sun et al. developed SERS‐hydrogel micropellets. The microdroplets were fabricated using a microfluidic chip through the combination of metal NPs with a PEGDA solution. The uniformly Ag‐loaded micropellets were employed for reproducible detection of glucose in the linear range 0.1–20 mm and with a LOD of 10 µM [88]. SERS‐hydrogel micropellets exclude the large proteins and fats in the blood, allowing the permeation of the small molecule glucose, enabling direct analysis of the biological samples without sample preprocessing. By requiring only microliters of blood and 20 min for complete measurement, the described platform offers the possibility of multiple detections in liquid biopsy. A different microfluidic approach was presented by Yue et al., developing an oil‐free microfluidic method for the fabrication of cleaner and versatile microparticles. In this study, Au NPs were immobilized in semipermeable alginate microparticles and employed for serum detection of 6‐thioguanine (6‐TG), an immunosuppressant drug commonly used to treat breast cancer, leukemia, and inflammatory bowel disease [86]. The recorded SERS spectra showed distinct spectral signatures when the semipermeable alginate microparticles were exposed to serum containing 6‐TG compared to serum alone. The SERS signal response was pH‐dependent and enhanced in acidic conditions. Compared to available methods, the hydrogel microparticles allow for fast and pretreatment‐free drug detection, showing promising potential in the therapeutic drug monitoring field (TDM).

#### 3.1.3 Template‐Removal System Method

This method involves the use of a sacrificial template that guides the formation and organization of plasmonic nanostructures during synthesis. The template provides a structural framework for nanoparticle assembly. Once the desired SERS‐active architecture is formed, the template is chemically or physically removed, typically through etching or dissolution, leaving behind a porous or hollow nanostructured scaffold composed of noble metals (e.g., gold or silver) (Figure 3C) [111, 112]. This approach enables the creation of complex, high‐surface‐area substrates with abundant SERS “hot spots” and tailored porosity, ideal for enhanced Raman signal amplification and analyte accessibility. Zhu et al. did a sequential template removal followed by a template‐persistent method for the final SERS substrate. On PS microspheres, silica nanocrystal‐embedded microspheres were synthesized. The PS template was then dissolved by toluene treatment to achieve a new template of hollow silicon nanocrystal embedded microspheres (SiPM). This template can reduce noble metal ions, and it was further mixed with HAuCl_4_ to provide AuNPs on the surface of the template. Through detection of 4‐mercaptopyridine (4‐MPy), the particles could sense pH‐changes in a wide range in PBS solution, down to concentrations of 10^−9^ m and with an EF value of 5.4 × 10^7^ [112]. In addition, Vikulina et al., demonstrated various methods for synthesizing Au microshell structures from a CaCO_3_ template removal as SERS substrates. Either by immobilization of AuNPs into the crystals by adsorption, by reduction of AuHCl_4_ in situ, or a combination of the two. Dried bovine serum albumin (BSA) was used to evaluate the SERS capabilities of the Au microshells. The SERS spectrum was then collected from a dispersion of dried BSA and porous SERS substrate, and an EF of 7.6 ± 1.6 × 10^8^ was achieved. Additionally, an EF of 2 × 10^8^ was achieved for Rhodamine B [89]. In a similar manner, Li et al., produced AgNP/AuNP microspheres, with a size of 4.25 ± 0.82 µm, through a Layer‐by‐Layer (LbL) method onto CaCO_3_. AgNPs were produced in situ via the silver‐mirror reaction, whereas AuNPs were adsorbed to the CaCO_3_. A minimum of 5 layers of NPs was shown to be required for stable shell formation. The capsules showed an EF of 10^6^–10^7^ for R6G, and by immobilization of different *E.coli* strains on the capsule surface, the capsules could be used to detect bacterial presence, enhancing the major peak by 10^4^–10^9^ [91].

Another example of clinical diagnosis lies in the detection of microRNA (miRNA), a small noncoding RNA involved in regulatory pathways, including development, apoptosis, and proliferation of cells. miRNA expression profiles are cancer‐type specific, making them an essential biomarker in modern anticancer therapies by monitoring disease progression, therapeutic response, and resistance [14]. Yang et al. present a SERS platform for sensitive detection of miRNA 155, fabricated by assembling stimuli‐responsive DNA microspheres and a duplex‐specific nuclease (DSN) amplification strategy. At first, toluidine blue (TB), as a Raman dye, and CaCO_3_ microparticles, as core templates, were combined to form the TB@CaCO_3_ composite. Using an LbL technique, a DNA network was built around the core, with a linker strand ssDNA L to cross‐link the oligonucleotide layers. Finally, in the presence of EDTA, the CaCO_3_ was dissolved, leading to TB‐loaded DNA microspheres. By introducing miRNA155 in the system, large amounts of ssDNA were produced. Because of the complementary ssDNA D and the linker ssDNA L, the microcapsule gets destroyed and releases TB, obtaining a strong Raman signal. With this design, miRNA 155 is rapidly detected in the linear range 1 fm–1 nm and with a detection limit of 0.67 nm. This work proposes a new strategy, based on programmable DNA microspheres, for biosensing and early diagnosis of cancer.

Microsphere‐based SERS systems offer significant promise for real‐time, multiplexed detection in biomedical applications. Their high surface area, tunable porosity, and capacity for uniform hot spot generation make them well‐suited for sensitive analyte detection, even at low concentrations. These platforms are also highly versatile, as they can be fabricated using various methods, ranging from bottom‐up self‐assembly to microfluidic templating, allowing researchers to tailor production approaches to the available infrastructure and resources. This flexibility may facilitate broader adoption across different lab settings. Future advancements in material engineering, such as the incorporation of stimuli‐responsive or multifunctional coatings, could enable in situ monitoring of dynamic biological or chemical environments and improve compatibility with portable or implantable sensing devices.

However, several challenges must be addressed for successful translation. Batch‐to‐batch reproducibility remains a significant hurdle, particularly for systems based on stochastic aggregation or complex synthetic protocols. Signal variability in complex media and limited stability under physiological conditions further complicate deployment. In biomedical contexts, biocompatibility is a key concern, especially given the reliance on noble metals.

Ensuring scalability, biocompatibility, and regulatory compliance will be critical for clinical and commercial success.

### 3.2 Microneedles (MNs)

MNs are emerging as a transformative technology in both dermal drug delivery as well as disease diagnostics and monitoring [113, 114, 115, 116, 117, 118]. In these applications, MNs penetrate the stratum corneum, the skin's outermost barrier. In the field of drug delivery, MNs enable transdermal administration of a variety of therapeutic agents such as peptides, nucleic acids, and vaccines in a controlled, minimally invasive, and often pain‐free manner. MNs can be designed to respond to various stimuli (e.g., pH, temperature, light, or electric fields), allowing for precise, on‐demand sampling or release of drugs [113]. In the field of diagnostics and disease monitoring, MN patches are being developed to extract interstitial fluid (ISF) or integrate biosensors for continuous monitoring of biomarkers such as glucose or lactic acid. [117, 118] These wearable systems offer real‐time data acquisition with enhanced user comfort, supporting chronic disease management and early disease detection. Most current MN designs, developed for diagnostic purposes, aim for in vivo sampling followed by separate in vitro analysis [119]. However, the application of MNs for disease monitoring and diagnostics becomes more attractive when both detection and analysis can occur simultaneously in vivo. One way to achieve this is to combine MNs with an SERS‐active substrate and, thus, significantly increase the Raman signal. SERS‐active MNs have been suggested and developed for various applications, including the monitoring of drugs, inflammatory markers, or other biomarkers in the ISF facilitated by measurements through the skin [15, 120, 121, 122, 123, 124]. Additionally, SERS‐active MNs have been proposed to track the development of melanoma by measuring relevant biomarkers [125, 126]. Representative examples of SERS‐active MN systems and their biomedical applications are summarized in Table 2.

**TABLE 2:** Representative MN‐based SERS systems toward biomedical applications.

Fabrication method	Material	Size (length]	SERS substrate	Sample matrix	Analyte/target molecule	Sensing performance	Refs.
Micromolding (PDMS)	PEGDA	700 µm	HDPM (micromolding)	ISF	Acetaminophen	0.45 µm	[128]
	PEGDA@MeHA	558.2 ± 14.4 µm	AuNPs (thermal vapor deposition)	ISF	Paraquat, cefazolin, nicotine	Paraquat: 0.1 ppb, cefazolin: 0.01 ppb, nicotine: 0.01 ppb	[123]
	PMMA	1000 µm	AgNPs‐4‐MPBA@Au (marangoni effect 3 phase self‐assembly process)	Skin	Tyrosinase	0.05 U/mL	[125]
	NOA@dopamine	600 µm	Au@Ag‐Pt (drop casting)	Skin	Tyrosinase	0.01 U/mL	[126]
	NOA61@PATP	1000 µm	Au NPs (immersion)	Skin	Superoxide anion radicals/ROS	0–480 ng/mL	[136]
	PMMA@dopamine	450 µm	Ag NPs (tollen method)	Skin	pH, redox potential, ROS	pH: range of 4–8, redox potential: range of 471–599.8 mV, ROS: range of 0–480 ng/mL	[15]
	NOA	300 µm	Au NRs (surface‐assisted nanoparticle immobilization)	ISF	pH	pH: range of 5–9	[12]
(Electro Deposition)	Ag needle	500 µm	Au coated (electrode position)	GBM tissue	GSH	0.037 mm	[131]
(Hydrogel coated)	PMMA, alginate hydrogel	600 µm	Au@AgNPs (growth solution)	ISF	MB, MTO	MB: 0.06 µm, MTO: 5 nm	[120]
(CO_2_ laser)	PSS, filter paper	650 µm	AuNR (ink writing)	ISF/serum	Rhodamine 6G	ISF: 0.05 µm, serum: 0.5 µm	[132]
(3D printing)	Photocureable resin	700 µm	Au nanoarrays (sputtering deposition)	ISF	Uric acid	0.51 µm	[121]
(Commercial)	PMMA	800 µm	Au NPs (seed‐mediated growth)	ISF	H_2_O_2_	1 µm (Better sensing ability than the reported H_2_O_2_ content which is above 10 µm)	[122]
	PMMA	800 µm	Au NPs	ISF	DTTC (3,3’‐diethylthiatricarbocyanine)	LOD: 10^−8^ m	[134]
	PMMA	800 µm	IS‐Au NPs	ISF	Pyocyanin (metabolite of *P. aeruginosa*)	LOD: 0.3 µm	[133]
	Stainless steel	NA (diameter: 260 µm)	Au nanoshells (Au@SiO_2_)	ISF	Reduction of resazurin to resorufin	—	[135]
(Vacuum molding)	PLA	1200 µm	AuNPs	Skin (agarose skin phantom)	Adenine, MB, R6G	Dried adenine: 0.068 ppm, aqueous adenine: 0.68 ppm, aqueous MB: 0.016 ppm, dried MB: 0.16 ppm, aqueous R6G: 2.4 ppm, dried R6G: 0.24 ppm, small molecule: below 200 ppb, bacteria: 10^2 ^CFU cm^−2^	[129]
(Compression molding)	PLGA@dopamine (PD)@HA	750 µm	Au nanoislands	Intradermal ISF (skin)	MB	1 nm	[130]

Abbreviations: PDMS: Polydimethylsiloxane. PEGDA: Poly(ethylene glycol HDPM: High‐Density Plasmonic MOFs (Metal organic frameworks) ISF: Interstitial Fluid MeHA: Methacrylated Hyaluronic Acid AuNPs: Gold Nanoparticles NPs: Nanoparticles ROS: Reactive Oxygen Species PMMA: Polymethyl methacrylate AgNPs: Silver Nanoparticles 4‐MPBA: 4‐Mercaptophenylboronic acid NOA: Norland Optical Adhesive Au@Ag‐Pt: Gold@Silver‐Platinum NOA61: Norland Optical Adhesive 61 PATP: Poly(2‐aminothiophenol) NRs: Nanorods GBM: Glioblastoma MTO: Mitoxantrone PSS: Poly(styrenesulfonate) PLA: Polylactic acid R6G: Rhodamine 6G PLGA: Poly(lactic*‐co‐*glycolic acid) PD: Polydopamine HA: Hydroxyapatite.

To turn MNs into SERS‐active substrates, a variety of fabrication strategies involving surface chemistry and nano‐structuring techniques have been employed, including thermal vapor deposition [123], Tollen's method [15, 124], Marangoni effect 3‐phase self‐assembly process [15, 125, 127], and Seed‐mediated growth [126]. Additionally, SERS‐active MNs have been directly fabricated by micromolding polymers together with a high‐density plasmonic metal–organic framework synthesized by the authors. [128] This approach produced an MN surface capable of generating a high density of electromagnetic hot spots.

The most common fabrication technique for SERS‐active MNs for biosensing is micromolding [12, 15, 123, 124, 125, 126, 128, 129, 130]. This technique is attractive due to cost‐effectiveness and simplicity, and can be combined with vacuum or compression to ensure complete polymer conformance to the mold's structure [129, 130]. Besides micromolding, other fabrication techniques, such as electrodeposition [131], CO_2_ laser cutting [132], and three‐dimensional (3D) printing [121] have been suggested. Finally, commercial MN patches can also be obtained from commercial suppliers and later functionalized for SERS biosensing [122, 133, 134, 135].

#### 3.2.1 Micromolding

**FIGURE 4:**
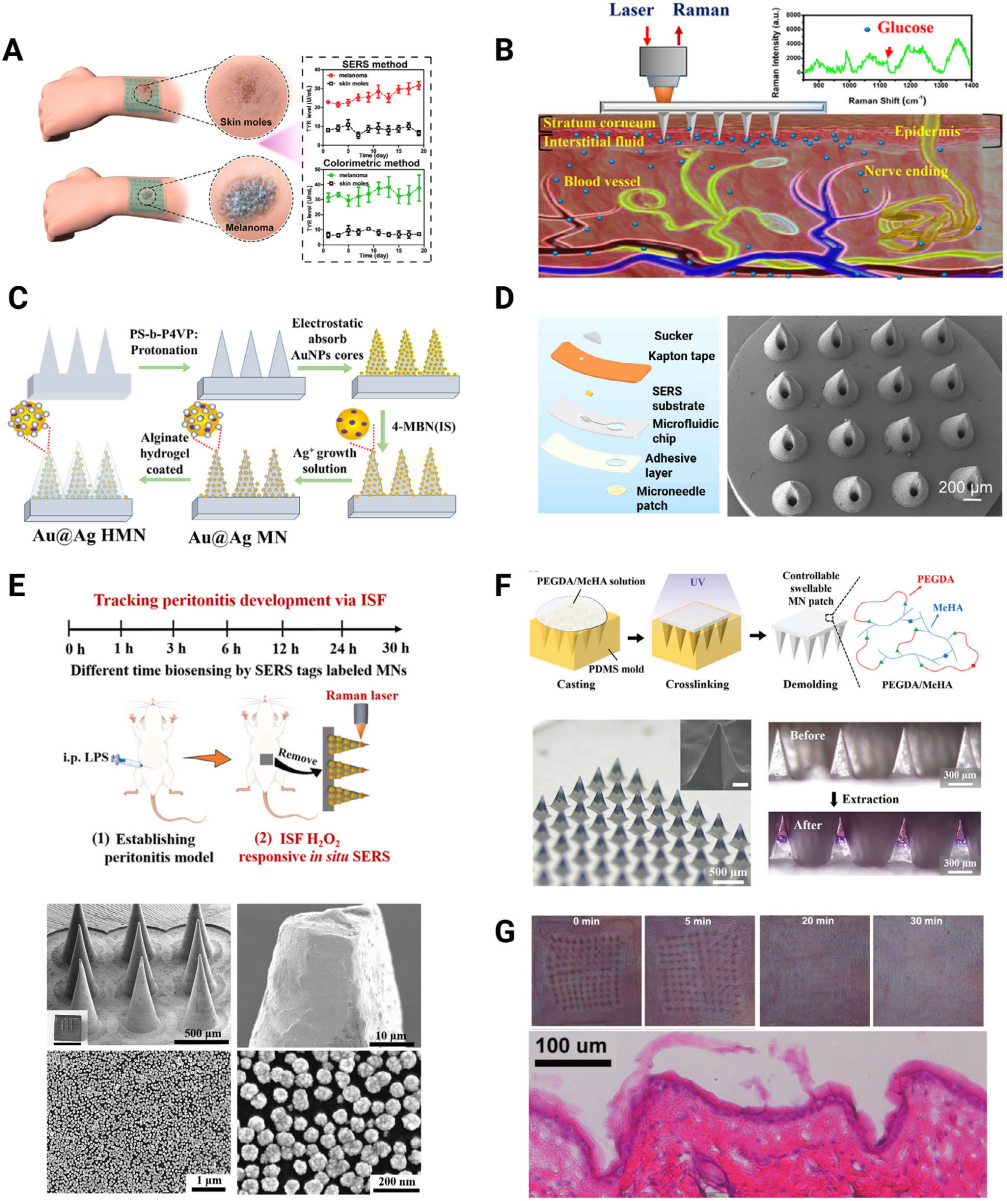
Fabrication and application of microneedle (MN)‐based SERS systems. (A) A wearable micromolded MN patch for dynamic monitoring of TYR levels for screening skin moles of healthy volunteers and melanoma patients. Reproduced with permission [126]. Copyright 2023, American Chemical Society. (B) Schematic illustration of glucose measurement in the interstitial fluid (ISF) using a PMMA MN patch fabricated by micromolding. Reproduced with permission [124]. Copyright 2020, American Chemical Society. (C) Schematic illustration of the fabrication process of hydrogel‐coated SERS MNs for drug monitoring in ISF. Reproduced with permission [120]. Copyright 2024, American Chemical Society. (D) Schematic drawing of a microfluidic‐based plasmonic MN biosensor fabricated using 3D printing, for uric acid ultrasensitive monitoring. The scanning electron microscope (SEM) image shows the hollow MN array. Reproduced with permission [121]. Copyright 2023, Elsevier Ltd. (E) Fabrication of SERS‐labelled MNs for tracking of periodontitis progression and treatment effect via ISF. The SEM images illustrate the MN array, the tip of a single MN, the high density and fewer aggregates of AuNPs distribution, and the core‐satellite shape of the AuNPs deposited on the tip. Reproduced with permission [122]. Copyright 2022, American Chemical Society. (F) Schematic illustration of the fabrication of MNs for a controllable‐swelling MN‐assisted paper sensing platform for personal health monitoring and optical images of the MN arrays before and after liquid extraction. Reproduced with permission [123]. Copyright 2023, Wiley‐VCH GmbH. (G) Reported microholes with a penetration depth of approx. 100 µm in the skin after application of SERS‐active MN patches, which disappeared after a maximum 30 min. Reproduced from [125] under the terms of the Creative Commons CC‐BY license.

Micromolding refers to a group of fabrication techniques used to replicate microstructures in polymers by utilizing molds to define the desired features [137]. The geometric characteristics of the MNs play a significant role in determining their properties, particularly in the fabrication process of polymer‐based MNs [138]. Hence, developing a scalable MN mold fabrication technique with precise control over the microstructure is essential for a wide range of MN applications. The most widely used mold material for MNs with SERS is PDMS [12, 15, 123, 125, 126, 128], but stainless steel has also been applied [124]. For the MNs themselves, various materials have been explored. One example is norland optical adhesive (NOA) [12, 126]. NOA is generally an interesting material as it enables the collection of a SERS signal through the MNs and can be applied for biosensing in situ, due to its transparency at higher wavelengths (e.g., 785 nm) and allows for functionalization of the polymer surface via its mercaptoester functional group [12, 126]. Additionally, the biocompatibility of various NOAs has been demonstrated as a culture scaffold for a large variety of mammalian cell types, including endothelial cells, fibroblasts, neurons, and stem cells without cytotoxic effects [139, 140, 141, 142]. Huang et al. [126] used NOA for micromolding of SERS‐active MNs to develop a wearable MN patch for the in situ evaluation of tyrosinase (TYR) levels in skin, using a SERS/colorimetric dual‐mode sensing for potential melanoma screening (Figure 4A) [126]. TYR is a key enzyme that exerts a regulatory influence on the synthesis of melanin, thereby playing a critical role as a biomarker for the detection of melanoma. The MN patch consisted of systematically arranged 15 × 15 arrays of MNs with a regular rectangular pyramid shape, placed 600 µm apart on the substrate. Each needle had a base diameter of 300 µm and a sharp tip extending to a height of 600 µm [126]. For SERS activation of the wearable NOA MN patch, the authors incorporated trimetallic Au@Ag‐Pt NPs. The trimetallic Au@Ag‐Pt nanorattles were made by a seed‐mediated method and a galvanic replacement reaction [126]. Seed‐mediated growth is a method of controlled NP synthesis where metal precursors are deposited onto the seed surface to create bigger or more complicated nanostructures based on small seed NPs [143]. On the surface of the Au cores, Ag shells were deposited by adding an AgNO_3_/ascorbic acid solution. After this, the galvanic replacement reaction was followed, which provided a uniform and regular thin Ag‐Pt shell at the Au@Ag surface [126]. The galvanic replacement reaction is a redox‐driven technique that creates hollow‐shelled or porous nanostructures by partially oxidizing a less noble metal, like Ag, and substitutes it with a more noble metal, such as Au or Pt [144, 145]. This offers extremely adjustable plasmonic NPs for SERS. This synthesized material is a critical part for the ultrasensitive SERS signal. Upon successful fabrication, Huang et al. [126] assessed the wearable MN‐based sensing platform for detecting TYR levels in human skin from volunteers. The MN patches were pierced into healthy skin, a suspicious abnormal skin mole, and in melanoma skin (Figure 4A). Here, the TYR expression was found to be slightly elevated in skin moles, while abnormally raised in melanoma patients. To gain further insight, measurements of TYR were performed in skin mole and melanoma volunteers at 2‐day intervals over a period of 20 days [126]. The obtained results confirmed that TYR levels of the melanoma patients were high and steadily increasing over time whereas the skin mole subjects displayed similar temporal TYR level profiles. However, TYR levels were not significantly different at different stages of melanoma. Overall, SERS‐active MNs show potential in melanoma screening, as a tool to assist general practitioners and dermatologists to differentiate melanoma from suspicious lesions and minimize the need for biopsies. This presents potential for early diagnosis, dynamic monitoring, and prognosis of melanoma.

As another diagnostic application, micromolded SERS‐active MNs have been proposed as a noninvasive tool for early detection and tracking of inflammation, by monitoring parameters such as pH, redox potential, and reactive oxygen species (ROS) levels. To this end, Peng et al. [15] used micromolding to fabricate multiplexed transparent SERS‐active MNs in polymethylmethacrylate (PMMA) for real‐time in situ detection of inflammation. Besides the strong mechanical properties of PMMA, it is particularly suitable for optical sensing applications due to its high light transmission property [15, 146, 147]. PMMA was cast into a negative PDMS mold, followed by UV polymerization for solidification. The MN array consisted of precisely structured needles, each with a base diameter of 250 µm and a height of 250 µm, optimized for efficient skin penetration and analyte detection [15]. To enable multiplexed SERS detection, the MNs were coated with a polydopamine (PDA) layer, which enhanced biocompatibility and provided strong anchoring sites for Ag NPs. AgNPs were then anchored on the PDA‐modified MNs via Tollen's method by adding AgNO_3_, NH_4_OH, and glucose solution. This method includes chemically oxidizing aldehydes to carboxylic acids while concurrently reducing Ag ions to metallic Ag. In the case of Peng et al. [15], the authors used a mild oxidizing agent, AgNO_3_ and NH_4_OH, as well as a glucose with an aldehyde group. The glucose was then oxidized into gluconic acid, which contains a carboxylic acid, producing an Ag mirror from the mild oxidizing agent. Subsequently, the authors modified the AgNP‐functionalized MNs with Raman‐active molecules, including 4‐mercaptobenzoic acid (4‐MBA), 8‐aminoquinoline (AQ), and 3‐mercaptopropyltrimethoxysilane (PATP). This functionalization enabled real‐time inflammation monitoring in healthy and inflammatory mice under deep anesthesia, via detection of pH, redox potential, and ROS levels [15]. The results confirmed significant acidization, an increase of redox potential, and ROS levels in inflammatory regions compared to healthy mice. To further evaluate the signal uniformity in mice, SERS imaging studies were also performed after sacrifice. The reconstructed images were consistent with the SERS microscopy results. The ROS level mapping of inflammation was not uniform, which was attributed to the physiological variations of the mice at room temperature over time after death. Still, significant differences were observed in the physiological environment, which proves the applicability of in vivo detection of the developed platform. The SERS results obtained were validated with commonly employed techniques such as micro‐electrode detection and ELISA/fluorescence image analysis, where comparable levels were observed [15]. This demonstrates a significant ability of SERS‐active MNs to detect complex inflammation in vivo, in situ, and in real time. This work is anticipated to provide ideas for initial screening of patients before clinical treatment, monitoring the progression of diseases, and evaluating the clinical treatment efforts.

Micromolded PMMA MNs have also been suggested for painless in situ intradermal glucose monitoring, as an alternative to the frequent painful testing diabetic patients usually undergo, to continuously monitor their glucose levels. For example, Ju et al. [124] developed a novel low‐cost SERS sensor for intradermal detection of glucose in ISF using painless MN patches (Figure 4B). The fabricated pyramidal MN array consists of a 10 × 10 arrangement, with each needle coated with a SERS substrate. Each MN had a base length of 200 µm and tapered to a sharp tip extending to a height of 500 µm, ensuring effective skin penetration for glucose measurement. The sensor integrates a 1‐decanethiol (1‐DT) glucose capture agent with Ag NPs to enhance surface Raman signals, making it suitable for minimally invasive glucose detection without the need for blood drawing. To protect the back surface of the casted PMMA MNs and preserve the high light transmission properties, an agarose gel layer was applied. As previously described for SERS‐activation of MNs, the MN array was then coated with a layer of PDA, followed by the deposition of Ag NPs using Tollen's method. The MNs were further functionalized by immersing them in a 1‐DT solution, which facilitated the capture of glucose molecules for detection. To evaluate the accuracy of the platform, the glucose levels were measured in three mice with induced diabetes and compared to a commercial glucometer, presenting no statistical difference between the two methods [124].

#### 3.2.2 Hydrogel Coating

A hydrogel, which is a hydrophilic polymer, absorbs and holds a large amount of water or biological fluids in its 3D porous network. Hydrogels are widely employed in biomedical fields for applications including drug delivery systems, scaffolds for tissue engineering, wound dressings, and biosensor applications, since they are adjustable, biocompatible, soft and elastic, and with a good permeability to oxygen and nutrients‐all qualities resembling our tissues. Hydrogels are a great option for a range of medical applications, facilitating faster healing and offering better patient care. Current research is actively looking for innovative strategies to include hydrogels in MN‐based SERS systems. These devices aim to extract ISF from the human body by utilizing the hydrogel's swelling capabilities, while also incorporating SERS‐sensitive NPs into the hydrogel matrix to enable SERS‐based detection, allowing for real‐time monitoring of medication levels.

As one example, Li et al. [120] introduced hydrogel‐coated SERS MNs for painless, real‐time drug monitoring in dermal ISF, offering an alternative to traditional blood analysis. PMMA MN arrays were commercially manufactured, and their surface was treated with a PS‐b‐P4VP solution, enabling hydrogen bonding between P4VP and PMMA (Figure 4C). This treatment facilitated uniform electrostatic adsorption of negatively charged spherical AuNPs, ensuring consistent detection [120]. Electrostatic adsorption is the technique by which charged particles, ions, or molecules are attracted and trapped to a surface with the opposite charge via Coulombic forces without establishing covalent bonds [148, 149]. 4‐Mercapto benzonitrile was then coupled to the AuNPs via an Au─S bond, creating an internal standard signal. In situ growth of AgNPs on the AuNPs resulted in a core–shell Au@Ag structure, which significantly enhanced the electromagnetic properties. This method creates and deposits material directly on the surface without isolating NPs from an alternative solution. The bimetallic Au@Ag core‐satellite NPs were expected to provide a stronger electromagnetic enhancement compared to traditional Au@Au NPs. Finally, a sodium alginate hydrogel coating was applied to the MN surface, to ensure rapid extraction of ISF, promote drug molecule adsorption on the SERS substrate, and prevent Au@Ag NPs from detaching during skin insertion [120]. To evaluate the performance of the platform, the SERS MNs were placed for 10 min on the back skin of mice intravenously injected with MB or MTO at clinically relevant concentrations. For drug monitoring applications, it is essential that the concentration measured in ISF is equivalent to the blood. To evaluate this, the authors compared the concentration of both drugs in ISF and serum. For MB, a larger AUC was obtained in ISF compared to serum (1114.8 and 489.8 mg/Lh, respectively), which was attributed to the slower drug clearance in ISF. In contrast to the data for MB, the concentration of MTO in ISF was dramatically lower compared to serum (AUC of 19.3 and 489.8 mg/Lh, respectively), and the clearance was faster (undetectable after 8 h in both ISF and blood). The authors concluded that the overall differences between the concentrations of the two model drugs in ISF and serum could be attributed to multiple factors, such as the molecular size, lipid solubility, plasma protein binding rate, and variability of the permeability of the capillary walls [120]. Overall, SERS MNs enabled the evaluation of the pharmacokinetics and distribution of two model drugs in ISF and blood, representing a promising alternative tool for assessing drug behavior in the body. However, the differences in concentrations between ISF and blood also highlight the importance of considering translatability when developing MNs intended to predict blood concentrations based on measurements in ISF. This aspect will require further investigation in the future to enable the implementation of SERS‐active MNs as an alternative to blood analysis.

#### 3.2.3 CO_2_ Laser Cutting

CO_2_ laser cutting utilizes a high‐powered CO_2_ laser to cut, engrave, or ablate a wide range of materials with high precision, excellent repeatability, and rapid speed. It provides a non‐contact process that is ideal for sterile or cleanroom environments requiring minimum contamination of the sample. Furthermore, biocompatible polymers such as PMMA, PDMS, poly(lactic‐*co‐*glycolic acid), and hydrogels can be easily shaped into complex configurations, allowing the creation of detailed microstructures for biomedical applications. By applying CO_2_ laser cutting, Kolluru et al. [132] proposed a flexible plasmonic paper MN patch for on‐patch detection of molecules in ISF. This system is low‐cost, label‐free, and optically tunable, making it an ideal solution for molecular sensing. The MN patches consisted of nine MNs, each 650 µm in length. Each needle had a base cross‐section of 50 × 150 µm, tapering to a sharp tip with a radius of curvature of less than 1 µm. A Whatman grade one filter paper strip, measuring 2 × 7 mm, was attached to the base of each MN patch, without covering the needles themselves. An ink writing technique was used to endow the MN patches with plasmonic properties. The poly styrene sulfonate (PSS)‐modified Au nanorods were incorporated into a clean ballpoint pen refill, and the refill was used to deposit the PSS‐Au nanorods onto a 1 × 7 mm filter paper. The plasmonic paper was then adhered, resulting in the creation of plasmonic paper MN patches. This design allowed for enhanced molecular detection capabilities with minimal invasiveness. To assess the utility of plasmonic paper MN patches, the pharmacokinetics of a model compound, R6G, were investigated in ISF and serum in rats intravenously induced with R6G. Besides, both spectra look similar; the intensity of the bands in ISF was lower than in serum. This can be correlated to the binding of R6G to plasma proteins, resulting in different concentrations between ISF and serum. The authors further explore this by evaluating the binding of R6G to isolated plasma proteins. A lower R6G concentration was observed in comparison to protein‐free serum samples, confirming that R6G strongly binds to plasma proteins and therefore may not partition well into ISF, resulting in lower concentrations. While many plasma proteins are also found in ISF, most of them, such as albumin, are found at lower concentrations due to the capillary membrane and interstitial barriers. These findings support the limitations described in the previous section and reaffirm the need for further research to improve the reliability of platforms based on ISF in predicting drug or biomarker concentrations in blood for diagnosis and monitoring.

#### 3.2.4 3D Printing

In recent years, additive manufacturing, also known as 3D printing, has advanced rapidly due to its ability to fabricate complex geometries, reduce material usage compared to traditional manufacturing processes, and offer greater design flexibility [150, 151]. Several methods exist for 3D printing polymer materials, including stereolithography, material jetting, material extrusion, and others [152, 153]. Among these techniques, photopolymerization has proven to be a practical approach for printing objects with micrometric or sub‐micrometric resolutions, offering high accuracy, fast printing speeds, and precise control over the dimensions of the desired object. Projection microstereolithography (PµSL) is a printing technique that constructs microstructures using an UV laser scanning system and a localized photopolymerization process. This method ensures high precision, reduces costs, and enables the use of various materials for biomedical applications [152].

Based on this fabrication technique, Xiao et al. [121] developed a wearable plasmonic MN patch for ultra‐sensitive label‐free detection of uric acid, a potential risk factor in kidney diseases, in ISF. The patch had a thickness of 300 µm, and consisted of an MN patch, an adhesive layer, a microfluidic chip, a SERS substrate, Kapton tape, and a suction cup (Figure 4D). The MNs (700 µm height) were uniformly conical in shape and neatly organized (4 × 4 arrays) on the substrate with a spacing of 900 µm. To enable SERS, an Au‐SERS substrate was fabricated by sputtering deposition of nanogold onto a silicon nanoarray. When evaluating the detection of uric acid, the authors verified the accuracy of the wearable plasmonic MN sensor versus commercial colorimetric assay kits and obtained a correlation coefficient of 0.998. Uric acid was quantitatively detected via its characteristic Raman peak at 640 cm^−^^1^ that originate from ring deformation vibrations, enabling sensitive analysis with a detection limit of 0.51 µm. Additionally, no obvious SERS responses to coexisting species, such as ascorbic acid, glucose, lactate, and urea, were observed when evaluating the selectivity of the MNs. By integrating a handheld Raman spectrometer, the platform successfully distinguished the uric levels in porcine skin with induced concentrations that simulate healthy and disease conditions [121]. The obtained results suggest the developed plasmonic MN sensor is a promising tool for kidney or general disease monitoring and diagnosis based on ISF sampling. However, further research is needed when observing the representation of uric acid concentration in ISF with respect to blood sampling, which is essential to evaluate the reliability of the platform, as variabilities can be observed in the distribution and the clearance of the compound.

#### 3.2.5 Commercial MNs

Finally, commercially available MNs in PMMA, [133, 134] or steel [135] have also been applied for SERS‐active MNs. For example, Mei et al. [122] developed an MN patch designed for the monitoring of hydrogen peroxide as a biomarker for early diagnosis of acute peritonitis and the evaluation of drug therapy effects (Figure 4E). The commercial PMMA MN arrays were covered with gold (Au) NPs in a two‐step process. In the first step, a PS‐v‐P4VP coating was applied to the raw PMMA MNs, allowing for the electrostatic adsorption of spherical Au cores (∼45 nm in diameter) [122]. Electrostatic adsorption is a simple process describing the technique of adhering pre‐formed cores or NPs onto a surface using electrostatic attraction. This electrostatic attraction utilizes the competing charges between the cores and the substrate [154]. In the second step, Au seeds were grown in situ on the Au core for 20 min [122]. With the help of a growth solution, the small NP seeds were further grown into larger or anisotropic structures such as rods, cages, and stars [155]. The amphiphilic P4VP layer facilitated the adsorption of high‐density AuNPs, while the hydrophobic PS layer ensured their uniform dispersion, forming a stabilizing corona. For the fabrication of the SERS‐labeled MNs, the MNs were further immersed in a 3‐MPBA solution, with 3‐MPBA acting as a sensing molecule for hydrogen peroxide. The final PMMA MN patch featured an array of 3 × 3 MNs, each coated with the SERS‐based substrate (Figure 4E). The MNs had a conical shape with a height of approximately 800 µm and a tip size of around 20 µm. These optimal dimensions allow for efficient access to the ISF, enabling a minimally invasive, painless skin puncture for diagnostic applications [122]. To evaluate peritonitis progression, sensor MNs were placed at different time points into the abdominal skin of mice for 15 min and then detached for in situ sensing by Raman equipment. A new Raman peak was observed at 879 cm^−1^ after 3 h of inducing acute peritonitis in mice, indicating the ability of the patch to diagnose the disease. The increase of the Raman peak at 879 cm^−^^1^ directly reflects elevated H_2_O_2_ levels in the ISF. Then, the hydrogen peroxide content in ISF was significantly elevated after 24 h of inflammation, with no further significant increase within 30 h, demonstrating stabilization of the disease at this stage. Similar studies in normal mice demonstrated that the MNs themselves did not contribute to an increased SERS signal of hydrogen peroxide due to a potential inflammation response after puncture. Finally, the platform was further investigated to track and assess the anti‐peritonitis effects of drugs (i.e., diclofenac or aspirin) during disease treatment. The hydrogen peroxide content in ISF was obviously reduced compared to the mice without treatment. This was supported by quantification of TNF‐α in blood, where similar results were obtained, and H&E staining of the tissue, where more infiltration of inflammatory cells was observed in the disease model compared to the treated groups and negative control applications [122]. Overall, the developed platform allows for monitoring different disease stages in diseases such as peritonitis, which includes the evaluation of the efficacy of drugs in the respective treatment. This is promising to serve as a universal sensing tool to enrich the variety and prospects of ISF‐based disease diagnosis.

#### 3.2.6 Biocompatibility

As highlighted in this section, SERS‐active MNs have shown promising potential as a biosensing tool for detecting and monitoring various drugs and biomarkers in animal models and humans, paving the way for future clinical applications. One recurring concern is that MN patches in direct contact with the dermis might have biocompatibility challenges and stability concerns, related to NP residues after removing the plasmonic MN patch. In addition, the structure of the MN patch may induce the scattering of laser light, thereby weakening the Raman signals [132, 156]. To minimize these physical and chemical biocompatibility issues, Hsieh et al. [123] developed a soft MN sensing platform based on hydrogels, instead of the traditionally applied plastic materials, integrated with assisted paper‐based sensing that offers ultrasensitive molecular recognition capacity (Figure 4F). A hydrogel‐based MN patch requires polymeric materials with high biocompatibility, excellent strength, and adjustable swelling behavior. Hyaluronic acid is a widely used swellable polymer due to its rapid and significant swelling capacity; however, its mechanical strength is insufficient [132, 157, 158]. By modifying the carboxyl groups of hyaluronic acid to produce methacrylated hyaluronic acid, the authors enhanced the stability of its internal network, thereby preserving its structural integrity [123]. Additionally, poly(ethylene glycol) diacrylate (PEGDA), a hydrophilic polymer with photo‐crosslinking capabilities, was used to enable precise shape formation while providing adequate mechanical strength for skin penetration and high light‐transmittance, which is beneficial for signal detection [123, 128]. Each needle had a base width of 297.5 ± 8.3 µm and an average height of 558.2 ± 14.4 µm [123]. The authors applied the fabricated MN patch on human skin to extract ISF, which was then transferred to the Au‐deposited plasmonic cellulose paper for SERS sensing. This cellulose paper was functionalized by thermal vapor deposition, a technique that enables the thin‐film deposition of various materials onto surfaces [159]. Cellulose paper is commonly used in a diverse range of analytical and diagnostic fields, offering multiple advantages such as simplicity, low cost, and environmental friendliness [160]. In their work, Hsieh et al. [123] illustrated the performance of the MN system by monitoring nicotine in three healthy, nonsmoking human volunteers. For that purpose, a nicotine patch was applied in the upper arm during three cycles of 20 min, presenting a 40 min gap between cycles. During this period, the MN patch was applied to the opposite arm 10 min before and after the nicotine patch was deposited. After removing the MN patch, the paper‐based SERS sensing platform was applied to monitor nicotine variations in ISF. It is notable to mention that, after retrieval of the MN patch, an array of microholes appeared on the human skin, revealing that the MNs could penetrate the epidermis and access the ISF. However, the fast healing of the skin (microholes became invisible after 10 min and the skin was completely recovered after 30 min), associated with no skin irritation or discomfort experienced by the volunteers, implies a low risk of potential infections and suggests the potential frequent use of the developed SERS‐active MN patches [123]. In most other cases, it has also been reported that microholes were observed in the skin after retrieval of the SERS‐active MN patches and that these holes disappeared after 7–30 min (Figure 4G) [121, 124, 125]. A penetration depth of 100 µm has been reported for SERS‐active MNs (Figure 4G) [125], indicating that they can pass through the stratum corneum and epidermis while minimizing interactions with blood capillaries and nerves, thus facilitating a painless application [161]. This rapid recovery suggests that SERS‐active MN patches are generally minimally invasive and confirms that the MNs only penetrate the epidermis of the skin, exhibiting minimal invasiveness, which is crucial in clinical applications. Additionally, integrating paper‐based sensing systems can be a potential pain‐free and minimally invasive sensing strategy for ultra‐sensitive, label‐free detection in clinical applications. However, the biosafety of SERS‐active MN platforms remains to be investigated after frequent use over a longer period. In the future, the potential for integrating SERS‐active MNs with biomedical electronics, such as self‐powered systems and smart patches, will further strengthen their dual role in therapeutic delivery and diagnostic monitoring, paving the way for future personalized and remote healthcare applications.

## 4 Dynamic SERS‐Active MNMs for Bio‐Sensing Applications

MNMs are an emerging technology capable of converting energy into mechanical motion at the micro‐ and nanoscale, offering innovative solutions to the limitations of traditional SERS platforms [19, 162, 163, 164]. They are usually divided into two main types depending on how they are powered: fuel‐driven and field‐driven. Fuel‐driven MNMs move by producing propulsion through chemical reactions that generate ionic or molecular flows [164, 165, 166]. In contrast, field‐driven MNMs rely on external physical forces, such as magnetic, acoustic, optical, or electric fields, to achieve motion [167, 168, 169, 170]. Because these two types of propulsion open different motion possibilities, MNMs can be designed in a wide range of shapes and materials to match the specific tasks of their movement and application. MNMs have progressed from simple experimental systems [164] to multifunctional tools with demonstrated potential in targeted drug delivery, diagnostics, treatment of cardiovascular diseases, wound healing, and environmental applications, including promising results in preclinical models [171, 172, 173, 174, 175, 176]. Significant progress has been made in fabricating MNMs for SERS sensing applications, including localized detection, active enrichment, improved signal sensitivity, and real‐time analyte tracking [11, 20, 177]. Representative examples of SERS‐active MNMs and their biomedical applications are summarized in Table 3.

**TABLE 3:** Representative MNM‐based SERS systems toward biomedical applications.

Materials	Shape	Size	Movement mechanism	Matrix	Target molecule	Sensing conditions	SERS performance	Refs.
SiO_2_@Fe@Au/Urease (inside)/aptamer MMs	Hollow nanosphere	3 µm	Urease‐driven self‐propulsion	Exosome suspension in 25 mm urea solution	Exosome	514 nm	35% better capturing efficiency	[183]
(Hierarchical structured MMs (Ag/SiO/Fe multilayer nanomembranes)	Tube	Length: 100 µm diameter: ∼12.18 µm	Magnetic field	Aqueous solution	Benzaldehyde (BA), Cu^2^^+^	532 nm, 1.32 × 10^6^ mW cm^−2^	fivefold for single enrichment, 19‐fold for coupled MMs	[184]
(Fe_3_O_4_@SiO_2_@Ag NMs	Rod‐like	1.3–2.3 µm	Magnetic field	Intracellular environment	Introcellular molecules (non‐specific)	514 nm	Significantly improved intracellular Raman signal	[184]
(Up‐converted nanoparticles @mSiO_2_‐TAPP/catalase@Au‐3‐MPBA NMs	Janus sphere	29.1–59.8 nm	Enzyme‐driven, photothermal self‐thermophoresis	Tumor microenvironment	H_2_O_2_	808 nm, 0.2 W cm^−2^	LOD: 10^−8^ m, linear range:100 nm–10 µm H_2_O_2_	[181]
(Carbon nanocoil (CNC)@Ni@Au NMs	Helical	10–35 µm	Magnetic field	HeLa cell cytoplasm	DAPI	633 nm, 2.4 mW	Subcellular level: detection of DAPI inside the cell nucleus	[181]
(Fe_3_O_4_@Au nanostars NMs	Core‐island structure	484 ± 69 nm	Nanozyme‐powered Self‐propulsion, magnetic field	Intracellular environment	Biomolecules inside HeLa cells	633 nm	83.48% increased after 60 min of treatment	[185]
(Si/Fe_3_O_4_/Au‐Ag MMs	Tubular	10–15 µm	Magnetic field	PBS solution, cellular environment (MDA‐MB‐231 cells)	DTNB	633 nm	Significantly enhanced SERS signal intensity	[186]
(Au nanorod/SiO_2_ /Ag NMs	Encapsulated nanowire	6.4 µm	Electrokinetic trapping	DI water	Uric acid, xanthine, hypoxanthine, guanine, adenine, adenosine monophosphate	532 nm, 360 µW	Strong Raman signal enhancement upon contact with the cell; signal loss at 15 µm distance	[187]
(Magnetic bead@Ag@Au MMs	Spherical microspheres	2.7 µm	Magnetic field	DI water	E. coli, *S. aureus*	785 nm, 0.438 mW	Identification and differentiation of E. coli and *S. aureus*	[188]
(Au NMs	Nanorods	50 nm	Ultrasound fields	DI water, DI water, human serum	R6G, DNA oligonucleotides, miRNA‐1246	633 nm	LOD: 10^−^^1^ ^3^ m for R6G and DNA oligonucleotides, 10^−^^10^ m for miRNA‐1246	[189]

Abbreviations: TAPP: 5,10,15,20‐tetrakis (4‐aminophenyl) porphyrin. DTNB: 5,5’‐Dithiobis(2‐nitrobenzoicacid).

Using precise control, MNMs can be navigated to chosen areas, where they latch onto cells and deliver their drug load in a timed, localized way [178]. SERS‐active MNMs stand out by overcoming the limitations of fixed SERS substrates, as they can move to precise locations, making targeted and efficient extracellular detection much easier. Traditional SERS setups utilize static substrates, which means the signal‐boosting spots remain in one place, making it challenging to target the correct spot, especially in complex environments [179]. But SERS‐active MNMs are different; they can move on their own or be guided right to where we need them. This means they can reach hard‐to‐access areas, such as tumors or inflamed tissue, and detect signals with significantly improved accuracy and sensitivity [180]. For example, certain MMs have been engineered with helical shapes and functionalized surfaces that allow them to be magnetically guided into individual cells [181]. Once inside, their structure enables them to physically penetrate cellular barriers, including the plasma membrane and even the nuclear envelope, with high precision. Coated with plasmonic materials such as gold, these MMs can then serve as nanoscale SERS probes, detecting biomolecular signals directly from the cytoplasm and nucleus with subcellular resolution. Dynamic concentration effects, photonic enhancements, and hot spot density influence sensitivity in SERS‐based MNMs.

Unlike static platforms, MNMs boost sensitivity through their autonomous or field‐driven movement, which increases the likelihood of analyte capture and enhances localized concentration at SERS‐active sites. One of the most effective ways to enhance SERS signal strength is by creating electromagnetic hot spots, often formed through nanoparticle aggregation. For instance, Ag NPs can grow inside porous silica‐coated wire nanoparticles, where dissolved PS beads leave behind gaps that bring the particles close enough to create strong signal‐enhancing hot spots. These silica coatings allow uniform nanoparticle coverage on complex surfaces and leave internal cavities that can carry and release molecules while enabling real‐time signal monitoring. Another strategy is to engineer specific patterns, like nanobowls or nanocaps, on the surface of nanomotors (NMs) [182]. Moreover, SERS signal intensity can be enhanced by inducing NM aggregation through external stimuli, such as light [58].

To better understand how SERS‐active MNMs can be optimized for biomedical applications, the following section introduces their propulsion strategies, including both autonomous and externally actuated approaches: light‐driven, electrically driven, magnetically driven, and ultrasound‐driven MNMs. Each propulsion mode contributes uniquely to the MNMs' ability to navigate complex biological environments and enhance the sensitivity and precision of SERS‐based detection.

### 4.1 Self‐Propulsion

**FIGURE 5:**
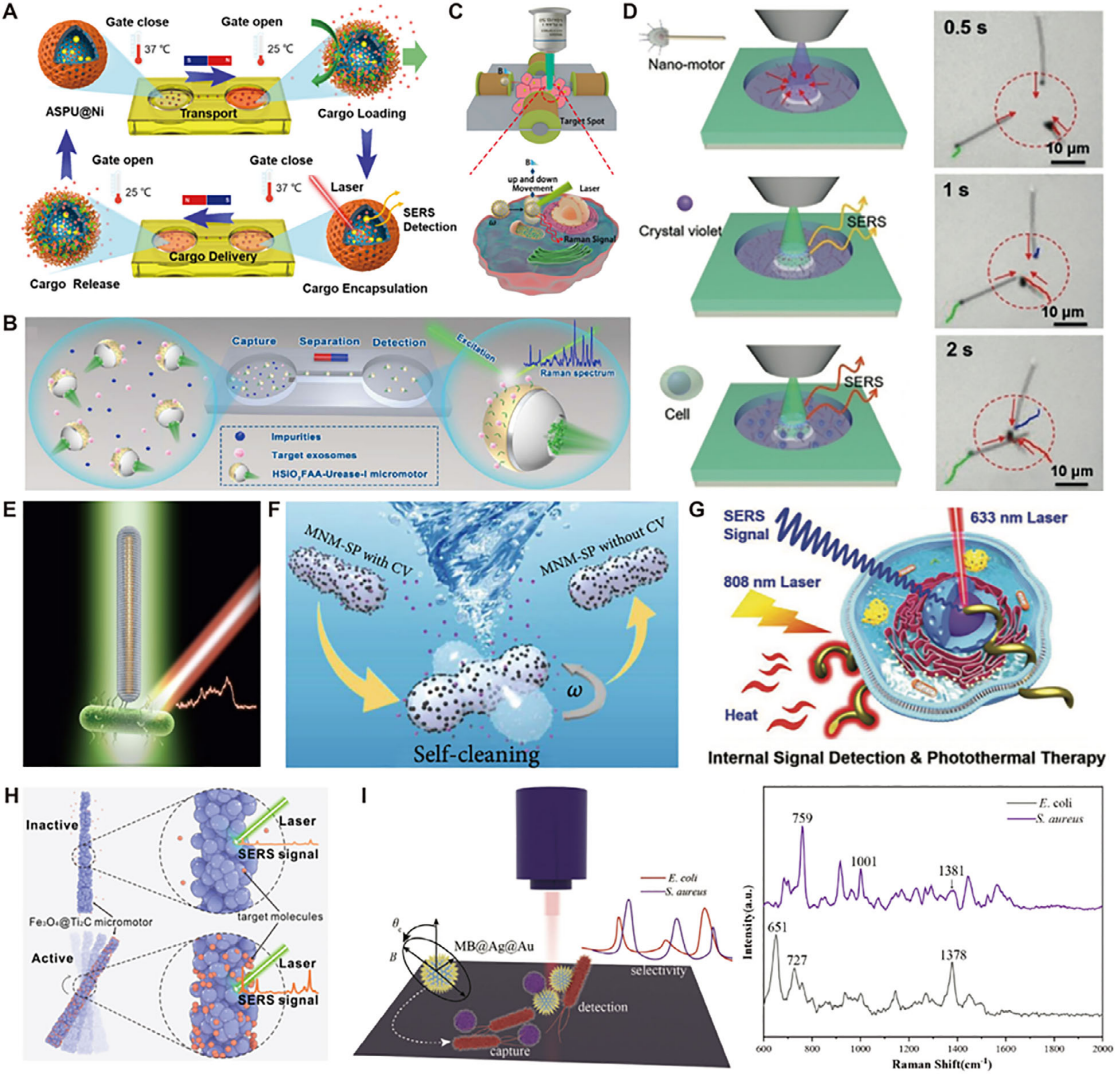
Applications of MNMs for SERS detection. (A) Schematic illustration of the sampling process from enzyme‐powered nanomotors (NMs), including targeting, loading, encapsulation, and release for SERS detection of crystal violet (CV) and rhodamine 6G (R6G). Reproduced with permission [197]. Copyright 2022, American Chemical Society. (B) Schematic of the process using aptamer‐modified MMs to capture and transport exosomes from a raw sample to a clean well for detection applications. Reproduced with permission [183]. Copyright 2023, American Chemical Society. (C) Schematic illustration of chemically driven, magnetically guided NMs for intracellular SERS sensing. Reproduced with permission [185]. Copyright 2023, The Royal Society of Chemistry. (D) Light‐induced aggregation for SERS detection and corresponding time‐lapse images showing the aggregation of NMs to increase signal intensity. Reproduced with permission [203]. Copyright 2018, Wiley‐VCH GmbH. (E) Intracellular SERS detection of the metabolite near an E. coli bacterium. The SERS signals changed as the NMs approached, contacted, and left the bacteria. Reproduced with permission [187]. Copyright 2023, Springer Nature. (F) Self‐cleaning capabilities of NMs for the SERS detection of various non‐covalent analytes. Reproduced from [184] under the terms of the Creative Commons CC‐BY 4.0 license. (G) Schematic illustration of SERS biosensing applications through targeted cell penetration with precise magnetic navigation. Reproduced with permission [181]. Copyright 2022, Wiley‐VCH GmbH. (H) Schematic of the molecular enrichment using magnetic rod‐like Fe_3_O_4_@Ti_2_C MMs to facilitate the SERS detection. Reproduced with permission [213]. Copyright 2024, American Chemical Society. (I) Ag nanoparticle‐doped magnetic MMs, followed by spiked gold shells, are used to capture and detect pathogenic bacteria using SERS. Reproduced with permission [188]. Copyright 2024, Wiley‐VCH GmbH.

Self‐powered MNMs move due to reactions between their surface and the surrounding environment. They are commonly referred to as chemically propelled micro/nano devices because they do not require additional external energy sources for propulsion. Materials such as Pt, Mg, Zn, MnO_2_, Ag, and biological enzymes are frequently used in fabrication [190, 191, 192, 193, 194]. To date, hydrogen peroxide (H_2_O_2_) has been the most extensively studied fuel, primarily for its ability to generate propulsion via bubble formation during its decomposition in the presence of Pt [195]. Compared to externally propelled MNMs, self‐propelled ones can achieve much higher velocities and greater power output. However, it is significantly more challenging to find suitable materials that are non‐toxic and acceptable for biomedical research. An essential application of enzymatic SERS‐active MNMs has been demonstrated before. Enzymes could act as biological analogs to catalytic metal surfaces and serve as engines for MNMs by converting chemical energy into motion using a wide range of substrate molecules [196]. For instance, Ma et al. synthesized core–shell structured NMs composed of Au@SiO_2_ nanoparticles coated with poly(N‐isopropylacrylamide) (PNIPAM), a thermoresponsive polymer, and further functionalized them with urease and nickel layers to enable catalytic propulsion and magnetic guidance [197]. The Au NPs (inside the hollow mesoporous silica shell) served as SERS‐based probes, and the PNIPAM polymer shell acted as a temperature‐sensitive gate. At the same time, urease enabled enzymatic propulsion by catalyzing the decomposition of urea into ammonia and carbon dioxide. This enzymatic reaction generated local chemical gradients, leading to the self‐diffusiophoretic motion of the NMs. They also achieved precise navigation and controlled sampling through magnetic guidance (via Ni coating) to specific locations. These NMs remain open below 31°C, allowing analytes to enter, but close at 37°C, effectively sealing their contents and preventing leakage or contamination during transportation. They were used for active molecular enrichment by encapsulating the molecules into the inner chamber, which contained the Au NPs for plasmonic hot spot generation inside, for real‐time detection (Figure 5A). The results showed that the Raman signals from inactive MNMs were 36% lower for crystal violet (CV) detection and 41% lower for R6G detection under the same conditions. These molecular enrichment strategies require only a minimal volume of solution, making them suitable for analyzing tiny sample volumes. In this study, CV and R6G were used as model analytes, and the system exhibited enhanced sensitivity through temperature‐gated uptake and urease‐driven propulsion. While specific LODs were not reported, the results highlight the potential of the platform for sensitive detection in small sample volumes. However, future work should address quantitative metrics, such as LOD, detection range, and analyte specificity, to evaluate real‐world diagnostic performance.

**FIGURE 6:**
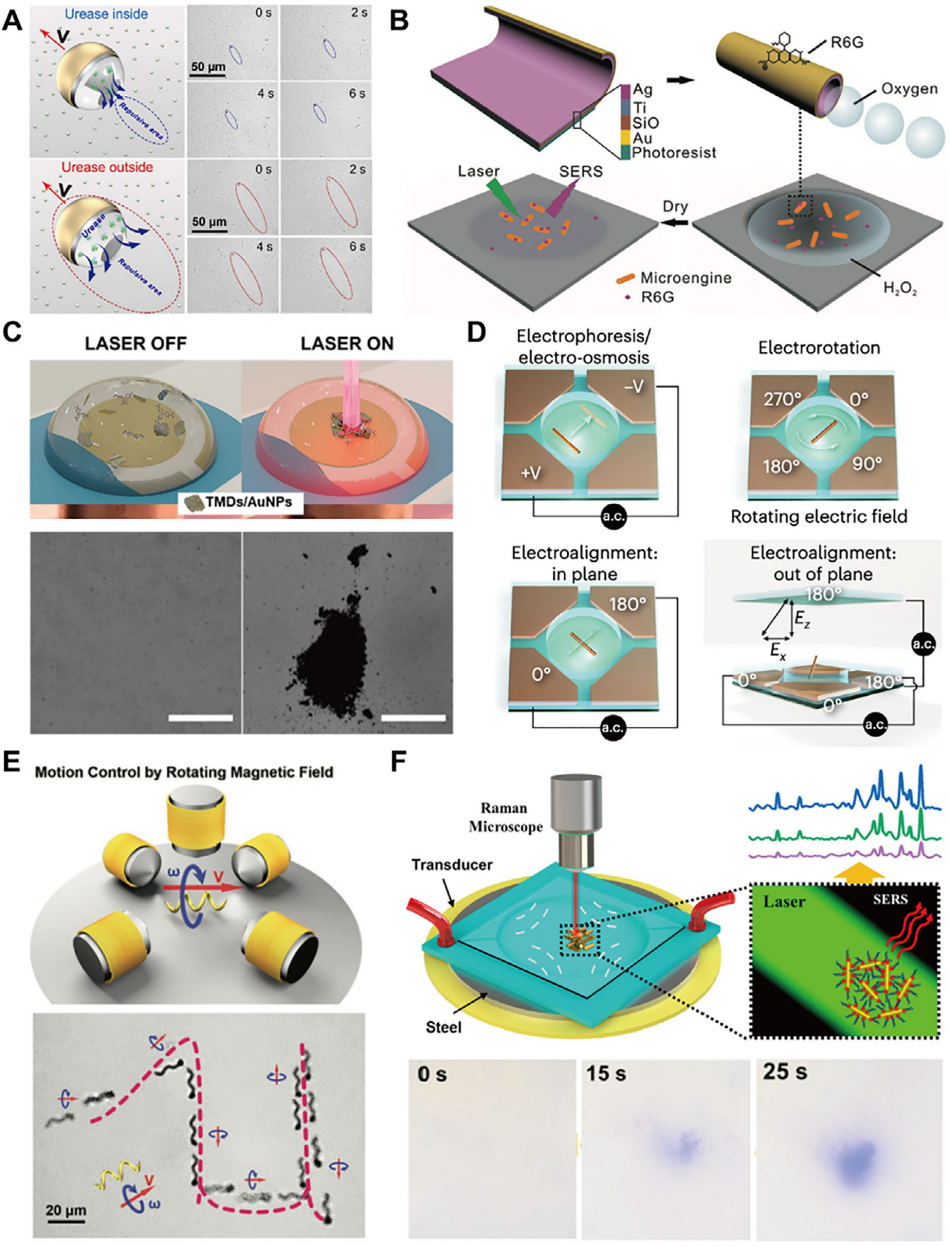
Methods for propulsion of SERS MNMs. (A) The enzyme‐driven MMs can be achieved by anchoring urease either inside (top panel) or outside (bottom panel) the MNMs, and the surrounding tracer particles indicate the repulsive area. Reproduced with permission [183]. Copyright 2023, American Chemical Society. (B) Illustration of a chemical‐driven rolled‐up Au/SiO/Ti/Ag MMs swimming in the H_2_O_2_ fuel by generating bubbles. Reproduces with permission [163]. Copyright 2016, The Royal Society of Chemistry. (C) Illustration of light‐driven MMs with photophoretic capability (top panel), and the swarm of MMs could be triggered and gathered by applying a laser. Reproduced from [58] under the terms of the Creative Commons CC‐BY 4.0 license. (D) Electrokinetic effects for driving NMs, including electrophoresis/electroosmosis and in‐plane and out‐of‐plane electro‐alignment for transport and orientation. Reproduced with permission [187]. Copyright 2023 Springer Nature. (E) Magnetically driven NMs using electromagnetic coils. Reproduced from [184] under the terms of the Creative Commons CC‐BY 4.0 license. (F) Illustration of the ultrasound‐propelled NMs with a transducer. Reproduced with permission [189]. Copyright 2020, American Chemical Society.

Similarly, urease has also been employed as a biocatalytic engine in larger MMs systems, such as hollow Janus silica‐based SiO_2_@Fe@Au/Urease MMs, where its localization (internal vs. external) plays a pivotal role in motion behavior and interaction with the surrounding environment [183]. Hollow Janus silica‐based SiO_2_@Fe@Au/Urease MMs were fabricated via a template‐assisted method, where PS particles were coated with SiO_2_, followed by Fe and Au sputtering, and template removal to yield a hollow structure with asymmetric functionalization. Subsequently, urease was selectively immobilized either inside or outside the hollow cavity using amine‐functionalized surfaces. While Au@SiO_2_@PNIPAM NMs combine enzymatic propulsion with thermoresponsive gating and internalized Au NPs for real‐time molecular SERS sensing, the SiO_2_@Fe@Au/Urease MMs demonstrate that directional electrophoretic propulsion and selective aptamer‐mediated exosome capture can also be coupled with SERS readouts (Figure 6A). They developed a strategy for the separation and SERS detection of exosomes from impurities by modifying aptamers on the surface of MMs (Figure 5B). The enzyme‐powered MNMs could swim around in the capture well and specifically trap exosomes due to the presence of aptamers, whereas the aptamer‐free MMs showed negligible specific adsorption. After capturing, the MMs with exosomes could be navigated using a magnetic field through a channel to a clear well for SERS detection. The platform achieved a capture efficiency of ∼80.7% for exosomes at an optimized urea concentration of 25 mm, which enhanced MM propulsion and contact frequency with the exosomes. Distinct Raman peaks corresponding to proteins, lipids, and nucleic acids enabled their subsequent label‐free identification. Compared to passive methods, the integration of mobility with aptamer specificity and plasmonic enhancement improves sensitivity and operational speed. However, challenges such as multiplexed detection, stability in biological fluids, and variability in exosome surface markers remain open issues for clinical translation.

Apart from urease, MNMs can also employ catalase as a propulsion engine, as demonstrated in a study, where Janus NMs were powered by catalase‐mediated decomposition of H_2_O_2_. [59] The NMs described in this work were synthesized from core‐shell upconversion nanoparticles (UCNPs) using thermal decomposition. These UCNPs were then coated with a mesoporous silica shell (mSiO_2_) to enable surface modification, followed by Au sputtering on one side to generate Janus asymmetry. The resulting asymmetric UCNP@mSiO_2_@Au NMs allow directional propulsion mechanisms. For propulsion, the catalase enzyme was conjugated to the surface, which decomposes H_2_O_2_ fuel to generate oxygen, driving motion via bubble propulsion and enhancing local oxygen availability for photodynamic therapy. Notably, the NMs were functionalized with a RR 3‐MBA which undergoes selective oxidation by H_2_O_2_, enabling SERS‐based quantitative detection of H_2_O_2_ in the tumor microenvironment. The system exhibited a linear Raman intensity response (*I*_882_/*I*_998_) over a detection range of 100 nm to 10 µm, with excellent sensitivity (*R* ^2^ = 0.998), suitable for detecting physiological H_2_O_2_ concentrations in tumors (typically ≤1 mm). By acting as both a chemical fuel and a detectable biomarker, H_2_O_2_ enabled NMs to move autonomously and perform on‐the‐spot sensing of tumor signals, enhancing both precision and therapeutic effect. Another example includes nanozyme‐powered magnetic NMs composed of Fe_3_O_4_ magnetic cores decorated with Au nanostar islands, forming a core–island architecture [185]. The Au nanostar possesses glucose oxidase‐like catalytic activity, stimulating glucose decomposition into gluconic acid and H_2_O_2_. The Fe_3_O_4_ core provides magnetic responsiveness, enabling directional navigation via an external gradient magnetic field. Using the plasmonic Au nanostar branches, they achieved highly sensitive SERS detection of CV down to 10 nm, with a linear response from 10 nm to 10 µm, and successfully captured intracellular biomolecular signatures (e.g., DNA, RNA) through magnetically enhanced analyte enrichment and hot spot‐driven amplification (Figure 5C). Bubble‐propelled MMs have also been studied for SERS detection [163]. First, multilayer nanomembranes (Au/SiO_2_/Ti/Ag) were deposited on a sacrificial polymer layer, and after dissolution, the pre‐stressed nanomembranes rolled up into tubular microstructures. Ag inside the tube catalyzes the decomposition of H_2_O_2_ into oxygen gas and water, and generated O_2_ bubbles are ejected from one end of the tube, propelling the MMs forward (Figure 6B). Pumera and his group synthesized rod‐like Au/Ag core–shell NMs capable of self‐propulsion in H_2_O_2_ fuel and highly sensitive solution‐based SERS detection of picric acid (PA) [198]. Although the study primarily focused on explosive detection, a ligand exchange was performed to replace cetyltrimethylammonium chloride (CTAC) with 16‐mercaptohexadecyl trimethylammonium bromide (MTAB). This modification enhanced the biocompatibility of the NMs, reducing cytotoxic effects and enabling their use in live cell cultures. Using MTAB‐stabilized NMs, the authors achieved a LOD of 100 nm PA in aqueous solution without any incubation period, enabling real‐time SERS sensing. However, the detection was conducted only in controlled aqueous solutions, and no testing in real environmental or biological samples was reported.

### 4.2 Light‐Driven Propulsion

According to Newton's third law, most light‐driven MNMs are propelled by creating a local asymmetric field that induces slip flow near their surface or by generating direct hydrodynamic forces. Light‐driven MNMs stand out by utilizing light as a tunable energy source, thereby eliminating the need for chemical fuels and offering advantages in control, efficiency, and biocompatibility [199]. Propulsion mechanisms include electrophoresis, chemiphoresis, thermophoresis, bubble generation, and photoresponsive deformation, focusing on slip flow driven by interactions between the MNM surface and the solution's double layer. Each propulsion mechanism operates through different interactions between the MNM surface and the surrounding solution, which plays a key role in how well the MNMs perform and how suitable they are for various environments. Electrophoresis and electrolyte diffusiophoresis rely on long‐range Coulomb interactions with the Debye layer, but their effectiveness decreases in high ionic strength conditions due to ionic screening. In contrast, nonelectrolyte diffusiophoresis and thermophoresis depend on short‐range interactions, making them more stable under such conditions, highlighting the “ion tolerance challenge” in MNM design. Many light‐powered MNMs use self‐electrophoresis or electrolyte diffusiophoresis for propulsion. In self‐electrophoresis, propulsion arises from separate anodic and cathodic reactions, creating an electric field. In contrast, in electrolyte diffusiophoresis, reactions occur on one side of the motor, and unequal ion diffusion generates the electric field that drives the motion [200, 201]. Au@MoS_2_ MMs functionalized with Au NPs were used for SERS‐based sensing, ensuring a uniform system due to collective photophoretic motion triggered by a 785 nm NIR laser, allowing for analyte preconcentration and enhanced detection of malachite green and paraquat in water samples (Figure 6C) [58]. The system's key feature is its ability to combine motion and sensing using a single NIR laser source, simplifying detection while avoiding external fuels or surfactants and enabling detection limits down to 0.1 µm for malachite green with high reproducibility (∼5%) and a linear response in the 0.3–10 µm range. In a complementary approach, Huang et al. created MMs by coating silica beads with Ag NPs, forming a dynamic and controllable SERS probe [69]. These AgNP‐coated silica beads were used as trappable MMs manipulated by dual optical tweezers integrated into a Raman microscope platform. Two such beads were trapped and precisely positioned to form a tunable plasmonic hot spot, which significantly enhanced the Raman signal and enabled sensitive detection of analytes, including proteins at physiological concentrations. The system achieved single‐molecule level sensitivity with a LOD of 100 nm for alpha‐synuclein. It demonstrated reproducible detection of hemoglobin at 100 nm and lysozyme at 1 µm in 1 × PBS buffer (pH 7.4), with RSDs of 14.6%–16.3%. In particular, the approach resolved subtle structural variations among transient species of intrinsically disordered proteins like alpha‐synuclein, an insight that is typically obscured in ensemble‐averaged measurements. Another study includes plasmonic NMs realized as Au nanotriangles (AuNTs), Au nanospheres (AuNSs), and Ag nanospheres (AgNSs) coated with a non‐photoresponsive cationic surfactant (CTAC), which endowed the particles with positive charge and facilitated light‐directed motion based on plasmon‐enhanced thermophoresis [202]. Upon low‐power laser illumination (532 nm, 0.03 mW/µm^2^) on a plasmonic substrate (Au nanoislands), localized heating induced a temperature gradient. This created thermophoretic motion, causing the positively charged nanoparticles to be driven toward the laser spot. Motion was reversible: switching off the laser led to disassembly due to electrostatic repulsion. In situ SERS was demonstrated by forming controlled “hot spot” assemblies for the detection of R6G (LOD ∼1 µm) and Methyl Orange (LOD ∼5 µm) under native aqueous conditions. While the study successfully demonstrates feasibility with model analytes, its practical applicability in real‐world biomedical or biochemical sensing remains to be validated.

To amplify Raman signals in localized nanoscale regions, Wang and collaborators developed light‐responsive, matchlike NMs, composed of a SiO_2_ core‐shell Ag nanowire (AgNW@SiO_2_) body and a rough AgCl spherical tail, forming a one‐dimensional asymmetric structure capable of self‐diffusiophoretic propulsion under UV light. The AgCl tail acts as a photocatalytic nanoengine, while the SiO_2_ shell enhances detection via shell‐isolated SERS (SHINERS), offering stability against oxidation and surface contamination. These NMs exhibit positive phototaxis, enabling light‐guided aggregation at the beam center, which significantly boosts local SERS signal intensity, achieving a 6.2‐fold enhancement for CV (10^−^^4^ m) and a threefold increase for MCF‐7 breast cancer cells compared to non‐aggregating counterparts (Figure 5D) [203]. The platform demonstrated sensitive detection of molecular and cellular analytes in situ, facilitated by active targeting motion and light‐induced clustering. However, challenges remain in ensuring uniform AgCl tail formation, precise control of shell thickness, and long‐term photostability for real‐world biosensing deployment.

### 4.3 Electrical Propulsion

Electric field propulsion is a widely used technique for manipulating MNMs [204]. Utilizing standard electrodes and power supplies, it can actuate a diverse range of nanoobjects, such as metallic particles, droplets, and microbubbles, through AC or DC fields. Propulsion mechanisms include electrophoresis, dielectrophoresis, electrowetting, and electroosmosis, with electric tweezers (quadrupolar microelectrodes) enabling precise two‐dimensional control. One of the first studies to combine electrically driven NMs with SERS features tri‐layer plasmonic nanocapsules composed of a metallic Ag/Ni/Ag nanorod core (enabling electric and magnetic manipulation), a silica middle layer (scaffold and quenching barrier), and an outer layer of Ag NPs (∼25 nm) providing high‐density SERS hot spots (∼1200 hot spots/µm^2^) [16]. Manipulation of these nanocapsules was achieved using “electric tweezers,” which employed orthogonally applied AC (∼15 V, 20 MHz) and DC (−2.5 to +2.5 V) fields for 2D alignment and transport with a spatial accuracy of approximately 150 nm. When illuminated by a 532 nm laser, the nanocapsules enabled single‐molecule detection of 1,2‐bis(4‐pyridyl)ethylene at concentrations as low as 1 pm, with an estimated EF of 1.1 × 10^10^ and spatially uniform Raman mapping (±9% signal variation) over the nanocapsule surface, as verified by confocal Raman microscopy. A similar triggering technique, using electric tweezers, was applied to the rotary nanomotors composed of tri‐segment Au/Ni/Au nanowires (acting as rotors), patterned magnetic bearings (Au/Ni/Cr), and quadruple microelectrodes (as stators) [205]. The combined AC (10–150 kHz, 8–17 V) and DC (−2.5 to +2.5 V) electric fields enabled precise 2D transport and orientation of nanowires with ∼150 nm accuracy, allowing them to be anchored magnetically onto nanobearings. Upon applying rotating AC electric fields with phase‐shifted voltages, the NMs achieved controlled rotation with tunable angle, speed (up to 18 000 r.p.m.), and chirality. The NMs' surfaces were coated with uniformly distributed Ag NPs for enhanced SERS sensing, forming dense plasmonic hot spots (<2 nm gaps). This system significantly amplified local electromagnetic fields, under 532 nm laser excitation, yielding an SERS EF of ∼10^10^ for MB and enabling single‐molecule detection down to 1 pm.

A different approach employed explored Au NPs, of approximately 20 nm in diameter, functionalized with Cytochrome c (Cyt c)‐specific aptamers and 4‐MBA to fabricate dual‐responsive plasmonic NMs capable of simultaneously sensing Cyt c release and cellular pH changes during apoptosis via SERS [206]. These NMs responded to external electrostimulation (0.3–1.2 V) applied via a conductive indium tin oxide (ITO) substrate, which triggered apoptosis and subsequent molecular release in adhered cells. This system demonstrated high sensitivity and selectivity, allowing differentiation between cancerous (MCF‐7 and HeLa) and normal (L929) cells based on the magnitude of Cyt c release and acidification. The SERS‐active NMs enabled real‐time in situ monitoring with a Cyt c detection limit down to ∼10 nm and a linear response from 10 nm to 10 µm. For pH sensing, the system demonstrated a clear linear correlation across the physiological pH range of 4.5–7.5. The selectivity was validated against a panel of potential interferents, including *L*‐glucose, vitamin C, Aβ 40, GSH, myoglobin, and others, showing negligible spectral interference. In terms of quantification, the triggered Cyt c release post‐electrostimulation was ∼1.4 µm in MCF‐7, 0.3 µm in HeLa, and <10 nm in L929 cells, while final cellular pH values shifted to ∼4.9, 6, and 6.3, respectively, demonstrating robust sensing performance with cell‐type specificity. These features make this SERS‐active nanorobot system a compelling platform for apoptotic mechanism studies and potential electrostimulation‐based therapeutic monitoring. Fan et al. successfully demonstrated a plasmonic nanowire‐based electrokinetic trap (Figure 6D) for the precise manipulation and SERS‐based detection of bacterial metabolites [187]. The nanosensors, constructed as SiO_2_‐coated Au nanorods (∼800 nm thick) decorated with ∼20 nm Ag NPs, enabled localized SERS detection of apoptotic biomarkers with high spatial precision. Raman spectra were recorded using 532 nm laser excitation at 360 µW, with an exposure time of 1 s per scan. Each spectrum was averaged over 20 consecutive scans to reduce random noise and enhance spectral clarity. Notably, Raman signals corresponding to metabolites (including uric acid, xanthine, hypoxanthine, guanine, adenine, and AMP) were only detected upon direct contact with single E. coli cells, with signal disappearance observed beyond 15–20 µm, indicating a strong distance‐dependent SERS response (Figure 5E). The system exhibited spatial positioning precision of 21 ± 3 nm and angular precision of 0.51° ± 0.04°, supporting real‐time tracking and orientation control of plasmonic nanoprobes with subcellular resolution. While a specific numerical LOD for Cyt c was not reported, the platform achieved label‐free, location‐specific detection with single‐cell resolution. It demonstrated exceptional SERS enhancement due to the dense Ag NP “hot spots” on the nanorod surface.

### 4.4 Magnetically Driven MNMs

Magnetically driven MNMs are propelled by magnetic fields and incorporate materials like Ni, Fe, Co, or their oxides to enable motion through either magnetic field gradients (magnetophoresis) or magnetic torque [207, 208]. These mechanisms enable MNMs to perform various motion types, including linear, rotational, corkscrew, and wagging motions, based on the field's orientation and strength. Gradient‐driven MNMs are drawn toward areas with stronger magnetic fields and don't need any special design adjustments. At the same time, torque‐driven MNMs rotate and align themselves with the direction of the magnetic field. Because the movement can be finely tuned by changing the type and strength of the magnetic field, this approach is beneficial for tasks that require precise control. MMs exposed to a magnetic field gradient naturally align their magnetic parts with the direction of the field. Once aligned, the gradient pulls them toward areas where the magnetic field is stronger, a process known as magnetophoretic motion.

An interesting example in this context was presented by Fan and colleagues, who developed bifunctional plasmonic‐magnetic (PM) nanotubes with tailored geometry and surface features for ultrasensitive SERS detection and magnetic manipulation [209]. These nanotubes were composed of a silica shell embedded with a Ni magnetic segment and uniformly coated with Ag nanoparticles on both inner and outer surfaces. Thanks to this dual‐layer plasmonic structure and tunable magnetic anisotropy, the nanotubes could be guided magnetically to individual live CHO cells for single‐cell analysis, enabling membrane‐level SERS measurements that revealed distinct lipid and protein signatures with single‐molecule sensitivity. Notably, no signal was detected from the cell surface in the absence of nanotubes, underscoring their essential role in enabling localized, ultrasensitive detection. In another example, rod‐shaped Fe_3_O_4_@SiO_2_@Ag NMs have been synthesized to move under a magnetic field gradient [184]. First, Fe_3_O_4_ nanoparticles (∼400 nm in diameter) were synthesized via a hydrothermal method. Then, an external magnetic field (0.9–3.5 mT) was applied to align the Fe_3_O_4_ nanoparticles into short chains due to their magnetic dipole interactions. A thin silica layer was grown on the surface of the chains to “lock in” the rod shape and provide chemical functionality. Ag NPs were deposited onto the silica surface through a silver mirror reaction. NMs have a permanent magnetic moment as they contain a magnetic Fe_3_O_4_ core (Figure 6E). When placed in a magnetic field gradient, they experience a magnetic force that pulls them toward regions of the stronger magnetic field, enabling remote‐controlled navigation. These NMs could remove the molecules from the surface within 30 min, as evidenced by the gradually decreasing Raman signals from the NMs (Figure 5F). This strategy indicated that the activated motion capability of the NMs could not only enhance the interaction with the analytes for rapid detection but also enable the recovery of the SERS NMs by removing the trapped analytes several times. While many SERS‐MNMs systems are developed for single‐use applications, making repeated measurements impossible, this study presents a significant advancement toward improved reproducibility.

Applying a rotating (or oscillating) magnetic field to MNMs can drive their movement, but this requires specially designed magnetic structures such as helical tails, flexible flat tails, or screw‐like shapes. The rotating magnetic field, which spins in a plane at a steady rate, must be oriented perpendicular to the MNM's rotation axis to effectively actuate these spiral or screw‐shaped MMs. For example, hierarchical structured MMs were prepared using a combination of nanoimprint lithography and rolling origami for integrated magnetic actuation and plasmonic SERS sensing [182]. First, the Al substrate was patterned with nanostructures (nanobowls or nanocaps) using anodic aluminum oxide stamps. A circular mask was applied to define the micromotor footprint, followed by sequential deposition of multilayer thin films (Au/SiO/Fe) using electron beam evaporation. These films were designed so that internal stress would cause the flat layers to roll up into tubular MMs when released from the substrate into ethanol, resulting in 3D structures with plasmonic surfaces for SERS sensing and embedded magnetic Fe layers for actuation. They were actuated using a rotating magnetic field. This motion significantly improved molecular enrichment at the SERS‐based surface compared to passive diffusion, resulting in stronger Raman signals and improved sensitivity. The SERS performance was markedly enhanced: Raman signals of R6G were detected down to 0.5 nm for the active MMs compared to 10 nm for the inactive ones, and the calculated enhancement factors reached ∼3.39 × 10^7^ for active MMs versus ∼1.17 × 10^6^ for passive ones, clearly demonstrating the power of active molecular enrichment in the motile hot spot, enabling ultrasensitive SERS detection. In a related study by the same research group, the previously developed concept of hierarchically structured MMs was extended by replacing Au with Ag as the outermost plasmonic layer, boosting the SERS performance [210]. While the initial Au/SiO/Fe MMs demonstrated excellent tunability in geometry and outer‐wall nanoarrays, the Ag‐based MMs were tailored explicitly for practical sensing applications due to Ag's superior plasmonic activity. Apart from Fe, Ni was employed to decorate carbon nanocoils (CNCs) due to its strong magnetic responsiveness, enabling magnetic actuation when exposed to a rotating magnetic field [181]. These CNCs were first synthesized via chemical vapor deposition, fragmented by ultrasonication, and then coated with Ni and a plasmonic Au layer via electron beam evaporation to create CNC@Ni@Au NMs. The helical shape and magnetic coating enabled corkscrew‐like propulsion, allowing for the targeted penetration of single cells for intracellular SERS sensing. Figure 5G shows magnetically driven helical NMs that could be actively navigated to a target cancer cell and penetrate both the plasma and nuclear membranes by the rotating mechanical force, enabling subcellular‐level discrimination, with SERS detection limits down to 1 nm and enhancement factors exceeding 10^7^, allowing distinct Raman signals to be obtained from the cytoplasm and nucleus for precise single‐cell biosensing. Fe_3_O_4_ nanoparticles are frequently chosen as a material for MM fabrication to endow the system with magnetic responsiveness. Combined with Ag NPs, they present an excellent platform for SERS‐based detection, where the rotation of hydrogel‐based MMs accelerates CV adsorption, enhancing the SERS signal [211]. Separately, silicon‐based MMs (SiMMs) modified with gold core‐silver shell nanoprobes and aptamers enable targeted cancer cell capture, free radical drug release, and dual‐mode (fluorescence + SERS) tracking under a magnetic field [186].

A different approach explored rotary MM sensors, fabricated using naturally occurring diatom frustules, which serve as robust, silica‐based microtubular scaffolds with periodic nanopores that function as photonic crystals [212]. These frustules were first purified via dispersion, sonication, filtration, and calcination, then coated on one side with a bilayer of Ni and Au by electron‐beam evaporation to enable magnetic actuation. Ag NPs were tightly packed on the diatom surface using a chemical reduction process, forming a uniform layer for improved SERS detection. Exposed to a rotating magnetic field, the MM‐sensors begin to spin in unison, reaching speeds of up to 1200 rpm, and gradually settle into individual microfluidic wells through guided self‐assembly. This system enables ultra‐sensitive (as low as 80 nm) and quasi‐real‐time SERS detection of fragmented double‐stranded salmon sperm DNA (average size < 2000 bp), achieving a fourfold improvement in capture efficiency and a threefold faster detection rate than static sensors.

Although MXenes are rarely used in magnetically actuated MNMs for SERS due to their reliance on chemical rather than electromagnetic enhancement, one study successfully integrated Fe_3_O_4_@Ti_2_C core–shell nanospheres into magnetically driven, self‐assembled rod‐like MMs capable of active molecular enrichment (Figure 5H) [213]. Leveraging charge transfer resonance, the system enabled selective enhancement for R6G, achieving a LOD as low as 10 nm. Improved sensitivity was also demonstrated for malachite green and fuchsin, down to 50 and 100 nm, respectively. The enhancement factor reached ∼1.05 × 10^4^ in the active state, compared to ∼1.2 × 10^3^ when inactive, demonstrating a nearly ninefold improvement via magnetic activation. Although MXene‐based MMs demonstrated excellent analytical performance for RR molecules, issues related to the chemical instability of MXene in aqueous environments and its potential cytotoxicity further limit this approach for real‐life biomedical applications.

However, a recent study showed that spiky gold‐coated microspheres (MB@Ag@Au) can serve as mobile SERS MMs for the selective detection and differentiation of pathogenic bacteria, specifically E. coli and *S. aureus* (Figure 5I) [188]. By navigating the MMs toward bacterial cells, using an external magnetic field, the system enables precise contact and real‐time identification through characteristic spectral fingerprints. While quantitative LOD values for bacteria were not provided, the MMs enabled clear spectral discrimination between E. coli and *S. aureus* based on characteristic purine‐related peaks. It is worth noting that the MNMs were mixed with only one type of bacteria at a time, meaning they could capture only a single bacterial type for SERS detection. Nevertheless, the same MNM system could be developed for detecting multiple bacteria by using different Raman tags or surface molecules that bind specifically to each type of bacteria. This would enable the identification of more than one target in the same sample.

### 4.5 Ultrasound‐Driven Propulsion

Ultrasonic propulsion is a promising method for MNM movement due to its safety for biological tissues and operational simplicity, with ultrasound typically generated by piezoelectric materials such as quartz or Lead zirconate titanate (PZT) [170, 214, 215]. Depending on how the quartz is cut, it can produce either body acoustic waves (BAW) or surface acoustic waves (SAW), each influencing MNMs differently. BAW enables particle trapping, like optical tweezers, while SAW generates radiation forces through leakage waves in the liquid. Most acoustic actuated MNMs rely on bulk acoustic streaming and standing‐wave fields, which require carefully engineered pressure nodes [216] (planes, rings, or spheres) for controlled manipulation, especially challenging in vivo. MNMs with asymmetric shapes and uneven density, such as concave rods, can generate axial fluid jets that drive fast motion, with propulsion direction even reversing at higher acoustic frequencies [217]. In this context, Wang et al. synthesized Au nanorods (which serve as MMs and SERS substrates] using a CTAB‐assisted seed‐mediated growth method and functionalized them with thiol‐modified DNA probes, allowing them to bind target DNA or miRNA by complementary base pairing (Figure 6F) [189]. The movement and aggregation of the Au nanorods were driven by an ultrasound field generated by a piezoelectric transducer. Ultrasound‐induced acoustic streaming at a mixing frequency (∼1.8 MHz) enhances the mixing and hybridization between DNA and nanorods. Standing acoustic waves created pressure nodes in the chamber at a lower frequency (∼620 kHz), causing the Au nanorods to move and aggregate at the center of the cavity due to acoustic radiation forces, creating effective hot spots. Ultrasound was a non‐contact, biocompatible tool that actively concentrated nanorods and associated analytes (e.g., R6G, DNA, miRNA‐1246) in a small detection zone, with ultrasensitive detection (down to 10^−^^1^ ^3^ m) due to the combined effects of ultrasound‐induced aggregated Au nanorod enrichment. Furthermore, ZnO nanorods coated with Ag NPs (ZnO─Ag) have been developed for sensitive SERS detection of targets like exosomes, DNA, and bacteria [218]. Ultrasound played a key role in generating acoustic streaming through a sharp‐edge acoustofluidic device, which actively mixed reagents and allowed for the controlled growth and Ag modification of the nanorods inside glass capillaries. This method facilitated the formation of well‐structured nanorod arrays, resulting in intense SERS hot spots for effective biosensing. The platform demonstrated remarkable sensitivity, achieving a LOD as low as ∼100 exosomes, 2.5 pm for single‐stranded DNA oligonucleotides, and ∼50 bacterial cells for E. coli, with even single‐cell identification capability. These results highlight the platform's ability to analyze targets across a broad range (nm–µm) with high specificity. While the acoustofluidic synthesis approach is rapid and cost‐effective, its reproducibility at an industrial scale and long‐term structural stability under physiological conditions require further validation. Additionally, its capillary integration into compact diagnostic devices with user‐friendly interfaces and robust sample handling remains a key hurdle for point‐of‐care translation. However, this approach was advanced by integrating acoustofluidic mixing and in situ synthesis of ZnO‐Ag nanoarrays within capillaries, enabling ultrasensitive, label‐free detection of exosomes at concentrations as low as ∼20 exosomes/µL. (219] The system achieved a broad linear detection range from 10^2^ to 10^8^ exosomes/µL with a LOD near 20 exosomes/µL, requiring only 0.5 µL of sample, demonstrating strong potential for point‐of‐care diagnostics.

Generally, MMs represent a promising frontier in SERS‐based biosensing, offering active transport, target enrichment, and localized signal amplification. Still, their practical deployment into integrated sensors will depend on advances in biocompatibility, navigation control, and integration into robust analytical systems.

## 5 Challenges and Perspectives

SERS has evolved into a compelling platform for specific, ultra‐sensitive detection in biological media. Over the past few years, advancements in plasmonic nanomaterials, micro/nanofabrication, and optical instrumentation have enabled numerous nano‐ and microscale SERS systems, ranging from static microspheres and MNs to dynamic MNMs. Different architectures demonstrate that SERS can be engineered for signal enhancement, spatial sensing, and operation directly in biological samples.

Microsphere‐ and microcapsule‐based SERS systems offer high surface areas, well‐defined hot spots, and tunable permeability for the detection of drugs, toxins, metabolites, and biofluids such as blood and serum. MN‐based SERS platforms extend these capabilities into the skin, enabling minimally invasive access to ISF for on‐site monitoring. Static pillar and MN systems generally offer reproducibility comparable to SERS microparticle and MM devices, which often exhibit greater variability in structure and performance, and thus in signal quality [15, 120, 122, 128]. Dynamic SERS‐active MNMs enable active transport and target enrichment, demonstrating that motion can be utilized not only for delivery but also to enhance sensitivity and precision. In our assessment, this diversity of form factors is a strength from a technology perspective, but it also makes standardization and regulatory efforts more complex.

From an engineering standpoint, this is an indication that SERS is not just a spectroscopy technique but is maturing toward a platform‐like technology that can be used for diagnostic device formats. SERS‐based point‐of‐care (PoC) devices offer fast, label‐free detection and quantification, which makes them well‐suited for identifying infectious or cancer biomarkers, and drugs in complex samples like saliva, blood, or urine. Moreover, they can be used for on‐site drug monitoring, tissue analysis during or after surgery, and minimally invasive skin diagnostics using microneedle sampling. The key advantage of the SERS‐based methods is their applicability in field applications. Therefore, for PoC applications, portable and handheld Raman instruments are essential to be paired with reliable substrates to make faster and more accurate analysis in clinical settings. For clinical use, these substrates should be simple, stable, easy to manufacture, provide strong and consistent signals, and capture targets quickly. Portable systems trade some sensitivity and resolution for speed and ease of use, so workflows should minimize sample preparation (e.g., simple filtration, microneedles, or lateral‐flow formats) and integrate automated sample prep, sample introduction, and on‑device data analysis to reduce user steps and variability. Finally, reproducible performance, substrate standardization, and clinical validation are required before routine deployment.

Despite the already successful in vitro and in vivo validations of biosensing applications, the implementation of SERS‐based diagnostic microdevices in clinical settings still faces several practical bottlenecks in comparison with established analytical methods, including chromatographic techniques hyphenated with mass spectroscopy. There is a trade‐off between cost, time‐to‐result, ease of use, and reliability. First, the variability of signals arising from local enhancement remains a barrier for deployment beyond research laboratories. This challenge is further emphasized when comparing results obtained in different laboratories. Very small variations in nanostructures and surface layers, as well as in optical conditions, lead to SERS results that differ, for example, between two laboratories. Increasing inter‐laboratory reproducibility requires standardized fabrication protocols and workflow, including clearly defined measurement conditions and benchmarking strategies, to enable comparison between different devices. Uniform and highly reproducible SERS probe preparation is crucial for ensuring repeatable detection [220]. In an engineering context, this translates directly into requirements for manufacturing, quality control, and performance specifications that can be met at scale, not just in single devices.

The sample introduction strategy and sample pretreatment requirements will affect performance for static SERS substrates. Blood or ISF can be directly applied to the surface (e.g., dipping or direct contact), which is user‐friendly and reduces the need for specially trained staff. Microfluidic perfusion strategies can enable automatic and even sample distribution over the detection area and are generally more reliable at scale [77, 80]. In contrast, microparticle‐ and micromotor‐based SERS devices only require homogeneous sample mixing, but their mobility could be a challenge to consistent localized detection [183]. From a device‐engineering perspective, we view microfluidic integration and physical confinement as key to enabling mobile SERS probes to function in a clinically usable manner. Although SERS offers molecular specificity, biofluids contain other components that interfere with analyte detection or saturate the probe surface. As a result, reliable quantitative calibration and linear response become very challenging, especially in multiplex detection scenarios. SERS systems must therefore either tolerate interference through surface chemistry and device design or rely on sample preparation and data processing pipelines that can be automated and validated [183]. The sample handling and pretreatment lead to safety and regulatory risks in relation to reliability and gold standards. Considering the variation in analyte levels between different sampling locations and matrices (dermal, serum, ISF), as well as the impact of sampling method and pretreatment, building robust correlations between SERS readouts and current gold‐standard analytical methods is essential.

Another consideration of regulations is biosafety, which will be just as important as sensitivity and limit of detection in relation to patient use. Metallic nanostructures are central to most SERS substrates, but their interaction with cells, tissues, and organs is still not fully understood. Studies on gold and other nanoparticles have shown that size, coating, and dose strongly affect toxicity, biodistribution, and clearance, which must be documented before clinical use [67, 68]. This is particularly critical for SERS systems that contact the body directly, such as MN patches or implanted probes, where there is a risk of metallic residue, local inflammation, or immune responses, compared with ex vivo or benchtop assays that only interact with the sample [119, 221]. In practice, invasive or transdermal SERS devices are typically regulated as medical devices. At the same time, non‐contact readers and consumables are more likely to follow in vitro diagnostic (IVD) routes, as they have different risk profiles associated with nanomaterial exposure to patients. Overcoming regulatory barriers requires that regulatory considerations be addressed in advance, already at the earliest stages of device design. Attention must be paid to the selection of materials, ensuring that only those acceptable from a safety and regulatory standpoint are employed. Device architecture must be aligned with the intended clinical use (whether the system involves direct patient contact or not), as this directly determines both the regulatory category and the risk level. Safety should not be demonstrated in a single step, but gradually through sequential validation, beginning with biocompatibility and followed by evaluation of stability, degradation, and toxicity, while final device performance should be compared with existing reference methods to demonstrate analytical comparability. This consideration of exposure and regulatory pathways, along with the consequent risk, should be part of the design process, not an afterthought.

## 6 Conclusions

The limits of Detection in SERS are sufficient in laboratory settings, so there is room and a need for the technology to mature into more robust systems and platforms. Current nano‐ and microscale SERS devices demonstrate that they can achieve clinically relevant sensitivity and specificity in biological media; however, what is still lacking is consistent performance in formats that can be mass‐manufactured, quality‐controlled, and utilized by non‐experts in the SERS field. In that sense, SERS has already proven itself as a sensing principle; the main challenge is adapting it to the users.

Across the reviewed architectures, the key challenges are reproducible substrate fabrication, controlled sample handling, and robust data analysis in realistic media, such as blood and interstitial fluid. For mobile systems, motion must be harnessed and constrained so that enrichment becomes an asset rather than a source of variability. At the same time, the biosafety of metallic nanostructures and early alignment with the correct regulatory route must be treated as primary design requirements, not afterthoughts.

Looking forward, we envision a promising path for highly integrated, application‐specific SERS platforms, featuring stable substrates, controlled microfluidics or sampling interfaces, straightforward user workflows, and automated, possibly machine‐learning‐assisted spectral analysis that yields clinically meaningful results rather than raw spectra. If these elements are combined with systematic clinical benchmarking against gold‐standard methods, SERS can move from proof‐of‐concept demonstrations to a realistic foundation for point‐of‐care diagnostics.

## Conflicts of Interest

The authors declare no conflicts of interest.

## Data Availability

Data sharing not applicable to this article as no datasets were generated or analysed during the current study.
